# A Simple Strategy to Eliminate Hexosylation Bias in the Relative Quantification of N‐Glycosylation in Biopharmaceuticals

**DOI:** 10.1002/anie.202002147

**Published:** 2020-07-09

**Authors:** Wolfgang Esser‐Skala, Therese Wohlschlager, Christof Regl, Christian G. Huber

**Affiliations:** ^1^ Department of Biosciences Bioanalytical Research Labs University of Salzburg Hellbrunner Strasse 34 5020 Salzburg Austria; ^2^ Christian Doppler Laboratory for Innovative Tools for Biosimilar Characterization University of Salzburg Hellbrunner Strasse 34 5020 Salzburg Austria

**Keywords:** bioinformatics, biopharmaceutical, mass spectrometry, monoclonal antibody, post-translational modification

## Abstract

N‐glycosylation may affect the safety and efficacy of biopharmaceuticals and is thus monitored during manufacturing. Mass spectrometry of the intact protein is increasingly used to reveal co‐existing glycosylation variants. However, quantification of N‐glycoforms via this approach may be biased by single hexose residues as introduced by glycation or O‐glycosylation. Herein, we describe a simple strategy to reveal actual N‐glycoform abundances of therapeutic antibodies, involving experimental determination of glycation levels followed by computational elimination of the “hexosylation bias”. We show that actual N‐glycoform abundances may significantly deviate from initially determined values. Indeed, glycation may even obscure considerable differences in N‐glycosylation patterns of drug product batches. Our observations may thus have implications for biopharmaceutical quality control. Moreover, we solve an instance of the problem of isobaricity, which is fundamental to mass spectrometry.

## Graphical abstract

**The sweet spot**: Quantification of protein N‐glycosylation variants by mass spectrometry is biased in the presence of glycation, a modification often observed in biopharmaceuticals. A novel computational method is able to eliminate this bias.

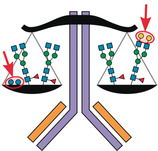

## Introduction

Protein N‐glycosylation arises from the enzymatic transfer of an oligosaccharide precursor to the side chains of asparagine residues followed by enzymatic remodeling of the N‐glycan structures.^1^ It commonly occurs on biopharmaceuticals and may influence their efficacy and safety.^2^ Hence, N‐glycosylation represents a critical quality attribute (CQA) of biopharmaceutical drug products.^3^ In the context of protein characterization and biopharmaceutical quality control, N‐glycans are routinely analyzed in their released, derivatized form by means of high‐performance liquid chromatography (HPLC) and fluorescence detection (FLD).^4^ In recent years, HPLC‐mass spectrometry (MS) analysis of intact proteins has increasingly been used in both academic and industrial environments.^5^ Qualitative and quantitative profiling of glycosylation variants (glycoforms) by intact protein mass determination is complementary to released glycan analysis: It retains the context of post‐translational modifications (PTMs) and provides information on pairing of protein subunits, as observed in dimeric antibodies.^6^ This is especially relevant in the context of regulatory issues if glycoform “fingerprinting” is to be considered as a CQA in the future.^7^

Yet, previous MS studies of intact glycoproteins have neglected the occurrence of different, isobaric PTMs, resulting from the attachment of a single monosaccharide to the protein. The most common of these modifications in biopharmaceuticals is glycation, which typically arises from a non‐enzymatic Schiff base reaction between the aldehyde group of a reducing monosaccharide and a primary amine on the protein (i.e., a lysine *ϵ*‐amino group or the N‐terminus), proceeding to a stable ketoamine via an Amadori rearrangement.^8^ Hexosylation may also arise from O‐mannosylation and O‐glucosylation, which have occasionally been observed on monoclonal antibodies (mAbs) produced in Chinese Hamster Ovary (CHO) cells.^9^ Glycation research has a long tradition in food chemistry, since heating typically induces considerable glycation of food proteins (e.g., lactosylation of whey proteins), which may reduce their nutritional value or even turn them into toxins or allergens.^10^ Glycation also plays a role in clinical chemistry: In diabetic patients, blood levels of glycated hemoglobin variant HbA1c (one glucose moiety linked to the N‐terminus of the β‐chain) are routinely monitored, since they provide information on average blood glucose levels.^11^ More recently, glycation has gained importance in the context of biopharmaceuticals, as this PTM may be induced both during production and storage of recombinantly expressed proteins. Specifically, high sugar levels (e.g., glucose or galactose) in the culture medium and in formulation buffers promote the formation of glycated species of the therapeutic protein.^12^ Importantly, glycation may induce aggregation of biologics, abolish their efficacy or even render them immunogenic, thus often necessitating its monitoring during production and storage.^13^

As attachment of an additional hexose results in a total protein mass gain isobaric to that of an N‐glycoform comprising a mannose or antennal galactose extension, these protein variants may not be discerned at the level of the intact protein. Therefore, relative quantification of N‐glycosylation based on intact glycoform profiles may be biased by the presence of glycation due to its isobaricity. Since no deglycating enzyme active against intact proteins is currently available, unbiased glycoform abundances are experimentally inaccessible by intact protein mass determination.^14^ To overcome this limitation, we here present a simple strategy to reveal actual glycoform abundances based on experimental determination of glycation levels followed by computational elimination of the hexosylation bias.

**Figure 1:**
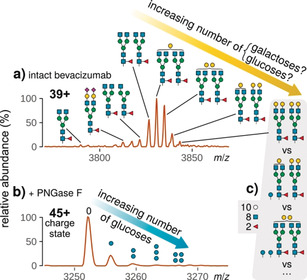
Raw mass spectra of a) intact bevacizumab at charge state 39+ and b) the same protein after de‐N‐glycosylation at charge state 45+. Assigned glycoforms or glycated proteoforms are drawn according to the Symbol Nomenclature for Glycans.^18^ Tilted arrows denote peak series whose zero‐charge masses differ by the mass of a single hexose. c) Alternative annotations for one peak in (a) with proteoforms whose monosaccharide compositions are identical.

**Figure 2:**
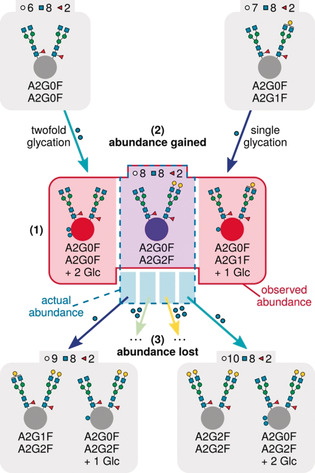
Contribution of different proteoforms to the observed abundance (1) of a mAb glycoform with the monosaccharide composition “8 Hex, 8 HexNAc, 2 Fuc”. Arrows indicate abundance gained from (2, top) and lost to other (3, bottom) proteoforms by means of glycation.

On the experimental level, the raw mass spectrum of an intact therapeutic mAb, such as bevacizumab, typically displays several peaks (Figure 1 a), each corresponding to a characteristic molecular composition which may arise from different isobaric proteoforms.^15^ In a previous study, we obtained mass spectra of an intact mAb upon analysis of fermentation samples in a dilute‐and‐shoot approach.^16^ Considering the known amino acid composition and possible N‐glycan structures, we were able to assign unique glycoform compositions for each peak using an in‐house software tool known as MoFi.^17^ Accordingly, the compositions associated with the five most abundant peaks contained two fucoses (Fuc), eight N‐acetylhexosamines (HexNAc), and six to ten hexoses (Hex); thus, the zero‐charge masses of these peaks differed by 162 Da. As enzymatic de‐N‐glycosylation of intact bevacizumab with PNGase F resulted in the detection of five signals, each differing by the mass of a single hexose, we concluded 1) presence of up to four glycations (Figure 1 b) and 2) existence of isobaric proteoforms differing in N‐glycan structures and number of glycations.^16^ For instance, while a peak could be annotated by the monosaccharide composition “10 Hex, 8 HexNAc, 2 Fuc” (Figure 1 c), it was impossible to directly quantify the underlying proteoforms, of which at least five exist, including A2G2F/A2G2F, singly‐glycated A2G1F/A2G2F, and doubly‐glycated A2G1F/A2G1F (mAb proteoforms generally comprise two N‐glycans, whose structures and nomenclature are explained in Table S1 in the Supporting Information). Hence, these isobaric proteoforms could not be distinguished by MS at the intact protein level.

These observations prompted us to develop a novel correction algorithm for indirect quantification of these proteoforms taking into account glycation levels (as determined upon enzymatic removal of N‐glycans by PNGase F^19^). While our previous work only reported preliminary results on elimination of the hexosylation bias as a side note, herein we provide an in‐depth description and validation of the correction algorithm. Moreover, we explore implications of the hexosylation bias for relative N‐glycoform quantification. To this end, we examined a panel of commercially available mAbs: Bevacizumab, a therapeutic mAb of subclass IgG1 that is expressed in CHO cells and has been approved for treatment of several types of cancer;^20^ National Institute of Standards and Technology monoclonal antibody reference material RM 8671 (NISTmAb), a recombinant humanized immunoglobulin of subclass IgG1κ expressed in murine NS0 cells;^21^ and Prolia^®^ (denosumab), a therapeutic IgG2‐type mAb that is produced in CHO cells and has been approved for the treatment of postmenopausal osteoporosis.^22^

## Results and Discussion

### Derivation of the correction algorithm from the glycation graph

Figure 2 exemplifies our algorithm for correcting the abundance observed for the glycoform with the monosaccharide composition “8 Hex, 8 HexNAc, 2 Fuc” (see the Supporting Information for details on the algorithm and its implementation). This observed abundance results from three factors (see numbers in Figure 2): First, a part of the observed abundance results from unglycated A2G0F/A2G2F (and unglycated A2G1F/A2G1F; Figure 2, center). Second, single glycation of the glycoform A2G0F/A2G1F (7 Hex, 8 HexNAc, 2 Fuc) yields a proteoform whose monosaccharide composition matches that of A2G0F/A2G2F. Hence, part of the abundance observed for the “8 Hex, 8 HexNAc, 2 Fuc” peak results from singly glycated A2G0F/A2G1F (Figure 2, upper right arrow). Similarly, doubly glycated A2G0F/A2G0F contributes to the abundance observed for A2G0F/A2G2F (Figure 2, upper left arrow). Third, single glycation of A2G0F/A2G2F yields a proteoform with a monosaccharide composition equal to that of A2G1F/A2G2F (9 Hex, 8 HexNAc, 2 Fuc). Hence, part of the actual abundance of the “8 Hex, 8 HexNAc, 2 Fuc” peak is lost to the A2G1F/A2G2F peak (Figure 2, lower left arrow). In the same manner, A2G0F/A2G2F loses a fraction of its abundance to the glycoform A2G2F/A2G2F (Figure 2, lower right arrow).

**Figure 3:**
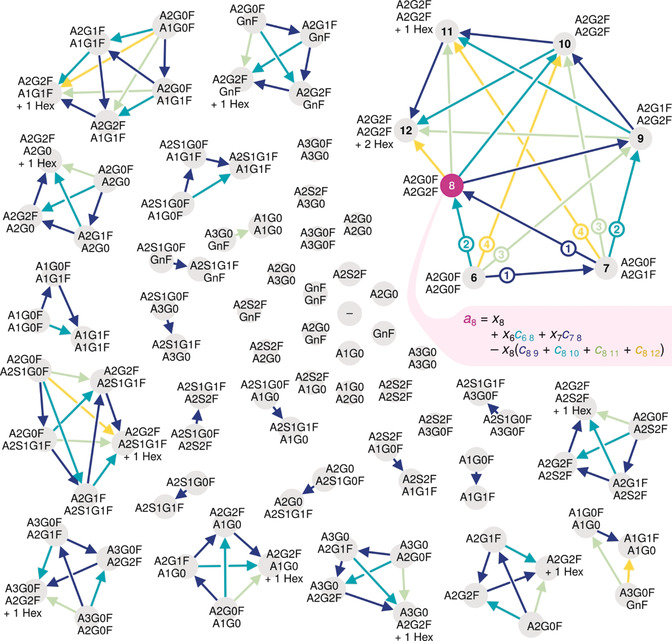
Glycation graph assembled from glycosylation and glycation data on bevacizumab. Each node (gray) represents a proteoform with a unique monosaccharide composition and is labeled by a glycoform that is compatible with this composition. Arrows link nodes whose monosaccharide compositions differ solely in their numbers of hexoses. Arrows emerging from nodes 6 and 7 (upper right) are labeled by those differences; all other arrows share this color scheme. (Node numbers in this connected component correspond to the number of hexoses in the respective proteoforms.) The version of Equation (1) (see Supporting Information) that applies to node 8 (pink) describes all the parameters that influence its observed abundance.

Evidently, simple subtraction of glycation abundances could not eliminate the hexosylation bias. Instead, we constructed a glycation graph to systematically gather all possible abundance transfers between proteoforms (Figure 3). In this graph, each node represents a monosaccharide composition, whereas each edge connects a pair of nodes whose monosaccharide compositions differ by one or several hexoses, but are identical otherwise. Hence, each edge indicates a possible addition of glucose moieties due to glycation. For example, the edge connecting the nodes representing the compositions “6 Hex, 8 HexNAc, 2 Fuc” and “7 Hex, 8 HexNAc, 2 Fuc”, respectively (i.e., nodes 6 and 7 in Figure 3), describes a single glycation in glycoform A2G0F/A2G0F, which yields a proteoform whose monosaccharide composition resembles that of A2G0F/A2G1F. Assembling such a glycation graph for bevacizumab revealed a considerable number of putative relations between glycoforms: Even this relatively simple glycoprotein (two N‐glycosylation sites, 14 distinct glycan structures) yielded a glycation graph containing 93 nodes (i.e., unique monosaccharide compositions) connected by 99 edges (Figure 3). The glycation graph proposed the following formula for determining the actual abundance *x_n_* of any monosaccharide composition *n* once the actual abundances *x_p_* of all monosaccharide compositions *p* with less hexoses have been calculated [Eq. (1)]:

(1) $$x_{n}=\frac{a_{n}-\sum_{p\in \mathrm{Pred}\left(n\right)}x_{p}c_{pn}}{1-\sum_{s\in \mathrm{Succ}\left(n\right)}c_{ns}}$$

(*a_n_*, observed abundance of node *n*; Pred(*n*) and Succ(*n*), predecessors and successors of that node, respectively; *c_pn_* and *c_ns_*, abundances of the glycation level associated with the edge from *p* to *n* and from *n* to *s*, respectively). Indeed, actual abundances could be readily calculated in the required order, since the glycation graph was directed and acyclic, and thus could be sorted topologically (see supporting information for details on the algorithm).

### Correction of glycoform abundances in bevacizumab fermentation samples

**Figure 4:**
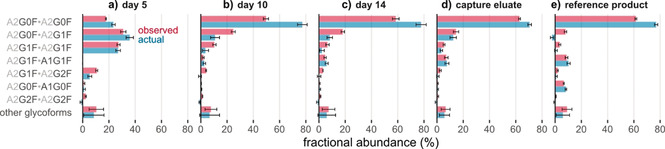
Glycoform abundances in bevacizumab fermentation samples (day 5, day 10, day 14), antibody purified via protein A affinity chromatography after 15 days of fermentation (capture eluate), and the reference product (Avastin^®^) before (observed) and after (actual) correction for the hexosylation bias. Error bars represent (propagated) 95 % confidence intervals from three technical replicates. See Table S1 for the abbreviations of glycan structures.

To demonstrate the functionality of our algorithm, we applied it to glycosylation patterns of bevacizumab in fermentation samples as determined by HPLC‐MS.^16^ Glycoform compositions and overall glycation levels were relatively quantified based on extracted ion current chromatograms (XICC) of intact and de‐N‐glycosylated bevacizumab, respectively. Comparison of corrected and observed glycoform abundances confirmed that glycation considerably impacts the latter (Figure 4). Notably, if glycation is ignored, abundances of glycoforms with fewer terminal galactoses (A2G0F/A2G0F and A2G0F/A2G1F) tend to be underestimated: Glycation masks their actual abundance by shifting their mass to values isobaric to glycoforms with additional terminal galactoses (e.g., A2G1F/A2G1F), which are therefore overestimated. For instance, correction increases the abundance of A2G0F/A2G0F in the sample drawn at day 10 of fermentation (Figure 4 b) from 49.6 % to 77.3 %.

Occasionally, the algorithm yields actual abundances that are negative, as observed for A2G0F/A2G1F in Figure 4 e (7.9 % before, −2.4 % after correction) and A2G2F/A2G2F in Figure 4 a (2.3 % and −1.2 %). While such negative values tend to occur for low‐abundant proteoforms and might be explained by measurement inaccuracies, they nevertheless challenge the validity of the correction algorithm. Importantly, elimination of the hexosylation bias makes one central assumption: It requires the probability of glycation to be equal for all glycoforms. Only if the glycation reaction is independent of the N‐glycan structures found on the protein, all same‐color edges in the glycation graph (Figure 3) will be associated with equal weights. (Notably, the correction algorithm does not impose comparable restrictions on the putative glycation sites, which may thus have different probabilities of glycation. Consequently, it permits the existence of so‐called glycation hot spots, which have been detected in several antibodies.12c, 19a, 23 Moreover, the probability for a given site may even depend on the glycation state of the remaining sites via allosteric interactions.) To examine whether glycation probabilities are indeed equal for different glycoforms, we considered a forced‐glycation study as a suitable method to test the validity of the correction algorithm.

### Forced glycation of NISTmAb

**Figure 5:**
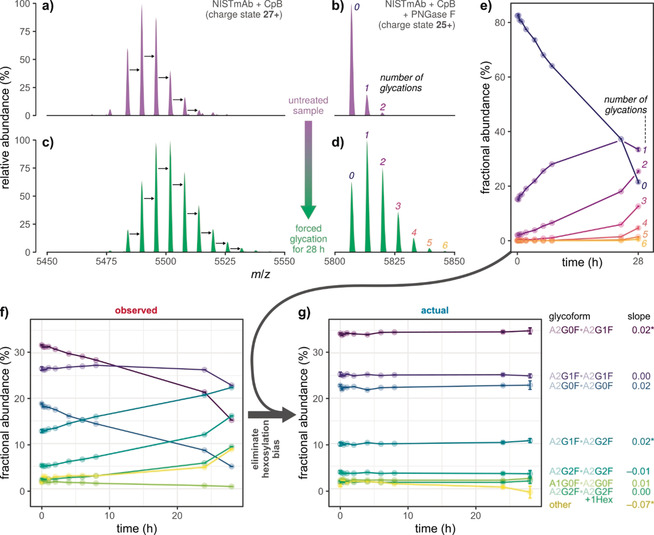
Apparent changes in glycoform abundances during forced glycation of NISTmAb. a)–d) Raw mass spectra of glycosylated and de‐N‐glycosylated protein in the untreated sample (top) and after 28 h of forced glycation (bottom), respectively. Black arrows denote mass shifts of 162 Da. e) Abundance of glycated species and f) observed glycoform abundances measured at ten time points during forced glycation. g) Actual glycoform abundances, as obtained by correcting values in (f) at each time point by the respective glycation level in (e). For each glycoform, the slope from a linear regression of actual abundance versus time is reported; stars indicate significance (*p*<0.05). All samples were digested using carboxypeptidase B (CpB) in order to remove heterogeneity caused by partial C‐terminal lysine clipping. Error bars represent (propagated) 95 % confidence intervals from five technical replicates.

To assess glycation induced under controlled conditions, we performed a forced glycation experiment using NISTmAb, a widely used reference material whose glycosylation profile has been extensively characterized in a comprehensive interlaboratory study.^24^ In agreement with the results of this study, the mass spectrum of intact, untreated NISTmAb displayed a characteristic series of peaks differing by 162 Da, respectively (Figure 5 a). Its five most abundant signals corresponded to glycoforms whose monosaccharide compositions are compatible with A2G0F/A2G0F and extended glycoforms with up to a total of four galactose residues. Removal of N‐glycans by PNGase F revealed that the bulk of NISTmAb was unglycated (82 %), while minor amounts of the protein were modified by one (15 %) or more hexose moieties (3 %, Figure 5 b).

Forced glycation via incubation with 500 mm glucose at 40 °C for 28 h induced a clear shift of abundances towards glycoforms with higher molecular masses. Moreover, new peaks emerged that retained the regular, hexose‐related spacing, which indicated that they represented multiply glycated proteoforms (Figure 5 c). In line with these apparent changes in the glycoform profile, species with up to six glycations were evident from the mass spectrum of PNGase F‐treated NISTmAb (Figure 5 d). To follow progression of the glycation reaction, we monitored glycoform and glycation abundances at several time points during the experiment (Figure 5 e,f).

The central requirement for the correction algorithm (i.e., equal probability of glycation for all glycoforms) predicts that any changes in observed glycoform abundances during forced glycation (Figure 5 f) were solely due to the hexosylation bias, which increased over time; actual glycoform abundances, however, should remain constant. Remarkably, elimination of the hexosylation bias confirmed that this was indeed the case (Figure 5 g). Moreover, the observation of negative actual abundances was negligible, implicating that the probability of glycation varied between glycoforms only to a minor degree. Hence, we considered our correction algorithm as a valid method for eliminating hexosylation bias in relative N‐glycoform quantification.

### Hexosylation bias in denosumab production batches

**Figure 6:**
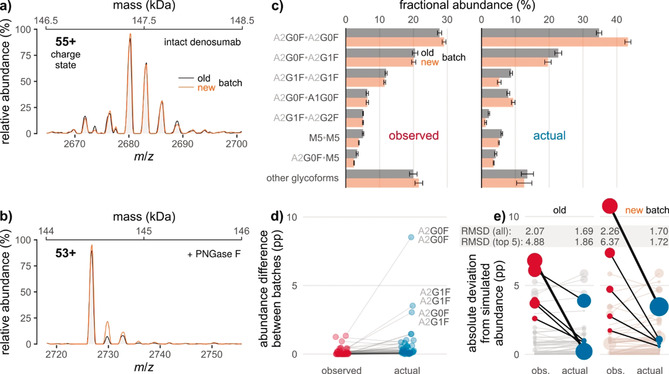
Glycation obscures differences between glycoform profiles of two Prolia^®^ batches (old vs. new). Raw mass spectra of a) the intact and b) the de‐N‐glycosylated mAb, corresponding to 2 kDa‐sections of the respective zero‐charge spectra (the secondary *x*‐axes indicate the respective masses). c) Fractional glycoform abundances before and after correction for the effects of glycation. d) Inter‐batch differences of glycoform abundances as derived from (c). Lines connect points denoting identical glycoforms. The three most common glycoforms are labeled. pp: percentage points. e) Absolute deviations of observed/actual glycoform abundances from simulated abundances based on released N‐glycan data. In each batch, those five glycoforms are highlighted for which correction leads to the largest decrease in deviation (thick line: most pronounced decrease). Point areas are proportional to observed/actual glycoform abundance. Error bars represent (propagated) 95 % confidence intervals from five technical replicates. RMSD, root‐mean‐square deviation.

Due to the observed effects of glycation on relative glycoform quantification, we hypothesized that glycoform profiles of biopharmaceuticals determined by intact protein MS may be obscured in the presence of glycation. Hence, we determined the glycoform and glycation levels of two different production batches of Prolia^®^. Mass spectra of the intact protein were almost identical, suggesting highly comparable glycosylation profiles of the two batches (Figure 6 a). Accordingly, glycoform abundances based on XICC quantification were highly similar (Figure 6 c, left chart). However, de‐N‐glycosylation revealed significantly different glycation levels of the two batches (Figure 6 b). Subsequent abundance correction by our algorithm indeed unraveled variation in the two glycoform profiles (Figure 6 c, right chart). In particular, abundances of the three most common glycoforms (i.e., A2G0F/A2G0F, A2G0F/A2G1F, and A2G1F/A2G1F) clearly disagree after correction (8.5, 3.0, and 3.5 %, respectively), but not beforehand (1.3, 0.4, and 0.4 %, respectively; Figure 6 d).

We also sought to demonstrate that corrected abundances outperform those observed, in terms of reflecting true values of relative N‐glycan abundances. To this end, we employed quantification of released and derivatized N‐glycans by means of HPLC‐FLD as an orthogonal analytical method that is not biased by glycation (Figure S1 in the Supporting Information). From this data, glycoform abundances were simulated by assuming random pairing of glycans, as previously described.19c, 25 Although statistical independence is arguable for pairing of mAb heavy chains,^26^ corrected glycoform abundances agreed more with the simulated values than observed abundances did, as judged from root‐mean‐square deviations (Figure 6 e). Notably, abundances of the most common glycoforms tended to display the most pronounced decrease in absolute deviation from the respective simulated abundances.

## Conclusion

In summary, we devised a highly innovative, interdisciplinary and generic approach for determining actual N‐glycoform abundances from HPLC‐MS data of intact glycoproteins, taking into account glycation levels. To this end, we combined three analytical strategies: 1) intact protein characterization by mass spectrometry to maintain the molecular context of PTMs and to unravel proteoform heterogeneity; 2) enzymatic dissection to resolve isobaric proteoforms; and 3) a computational method facilitating quantification of isobaric proteoforms at the intact protein level. We have ensured that the computational method is easily accessible for researchers from a non‐technical background. To this end, we provide both an intuitive graphical user interface for the correction algorithm, as well as a command line interface for integration in data analysis pipelines; both interfaces are extensively documented. Our algorithm will accept a broad range of input data, for example, abundances calculated from peak areas of a single mass spectrum or from XICCs. Furthermore, while we specifically focused on the ambiguity resulting from isobaric glucose and galactose moieties, the algorithm may correct for abundance effects of any PTM that is isobaric to N‐glycan subunits, such as core 1 O‐glycans, which are isobaric to poly‐N‐acetyllactosamine units on N‐glycans.

Despite the broad applicability of the correction algorithm, there is one important limitation: Measurement of glycation abundances requires successful MS analysis of the de‐N‐glycosylated target protein. In the case of the presented mAbs, N‐glycans could be readily released by PNGase F. Yet, other proteins might only be sufficiently stable or ionizable for MS analysis in the glycosylated state.

While we have focused on eliminating hexosylation bias introduced by glycation, it should be emphasized that other PTMs characterized by a mass shift of +162 Da are occasionally observed, such as O‐mannosylation and O‐glucosylation. Even though our own peptide‐mapping data corroborates the absence of these PTMs on the antibodies investigated (not shown), they might be relevant for other biotherapeutics, such as mAbs expressed in yeast.^27^ Notably, the correction algorithm will also work in the presence of O‐linked hexoses, since glycoform abundances will be biased in the presence of any hexose linked to the protein, irrespective of its chemical nature and site of attachment.

To our knowledge, our study is the first not only conceding that glycation levels influence relative quantification of N‐glycosylation variants, but also actually correcting for them: By applying the algorithm to therapeutic mAbs, we demonstrated that glycation may indeed conceal true glycoform abundances. This hexosylation bias is of particular relevance in the context of biologics, where N‐glycosylation profiles represent a CQA. Hence, our findings may have implications for biopharmaceutical quality control, since batches whose proteoform profiles are ostensibly identical may actually contain drastically different glycoform profiles after the effects of glycation on glycoform abundances have been eliminated. Indeed, the evaluated applicability of the computational method,^16^ combined with its simplicity, will allow implementation in an existing analytical setup both in academic research and in the biopharmaceutical industry. The generic approach described is extremely fast and informative, representing an attractive alternative to conventional targeted analysis of glycation or released glycans in both industrial and academic settings. Thanks to these assets, our method may readily be integrated into the clone‐selection process, the in‐process control in mAb production, as well as for batch‐to‐batch analysis of originator molecules or comparability studies of biosimilars and their respective reference drug products.

## Conflict of interest

Novartis BTDM, Sandoz GmbH as well as Thermo Fisher Scientific provide financial support for the Christian Doppler Laboratory for Innovative Tools for Biosimilar Characterization. The salaries of Wolfgang Esser‐Skala and Therese Wohlschlager are fully funded; Christian G. Huber's salary is partly funded by the Christian Doppler Laboratory for Biosimilar Characterization. The authors declare no other competing financial interest.

## Supporting information

As a service to our authors and readers, this journal provides supporting information supplied by the authors. Such materials are peer reviewed and may be re‐organized for online delivery, but are not copy‐edited or typeset. Technical support issues arising from supporting information (other than missing files) should be addressed to the authors.

Supplementary
