# Supplementary material for: A Simple Strategy to Eliminate Hexosylation Bias in the Relative Quantification of N‐Glycosylation in Biopharmaceuticals
Source: Angew Chem Int Ed Engl. 2020 Jul 9;59(37):16225–32. doi: 10.1002/anie.202002147 (PMC7539909; doi:10.1002/anie.202002147)
Supplement: Supplementary file 1 — Supplementary [file ANIE-59-16225-s001.pdf]

Angewandte Chemie
Eine Zeitschrift der Gesellschaft Deutscher Chemiker

Supporting Information

A Simple Strategy to Eliminate Hexosylation Bias in the Relative Quantification of N-Glycosylation in Biopharmaceuticals

*Wolfgang Esser-Skala, Therese Wohlschlager, Christof Regl, and Christian G. Huber**

anie_202002147_sm_miscellaneous_information.pdf

Contents

Experimental section *3*
Correction algorithm: Details . . . 3
Correction algorithm: Implementation . . . 4
Materials . . . 4
Identification and relative quantification of mAb glycoforms . . . 4
Released *N*-glycan analysis . . . 5
Forced glycation . . . 5
Code and data availability . . . 6

Supplementary figures *7*
Supplementary figure 1 . . . 7

Supplementary tables *8*
Supplementary table 1 . . . 8

Supplementary files *9*
Supplementary file 1: `cafog_source_code.zip` . . . 9
Supplementary file 2: `data.zip` . . . 9

Code for data analysis and figures *11*
Figure 1: Glycosylation and glycation . . . 12
Figure 2: Observed vs. actual abundance . . . 14
Figure 3: Glycation graph . . . 14
Figure 4: Quantification of bevacizumab glycoforms . . . 14
Figure 5: Forced glycation of NISTmAb . . . 17
Figure 6: Hexosylation bias in denosumab . . . 26

Session info *41*

References *44*

Experimental section

Correction algorithm: Details

Consider a monosaccharide composition n, containing a certain number of hexoses Hex(n). Three factors determine the abundance a_n observed for this composition (see Figure 2 in manuscript):

1. A part of a_n results from the unglycated glycoform(s) that can be assembled from n. We will call this portion the actual abundance x_n. For instance, a part of the abundance observed for the peak annotated with “8 Hex, 8 HexNAc, 2 Fuc” results from unglycated A2G0F/A2G2F (and unglycated A2G1F/A2G1F).
2. Any monosaccharide composition p which comprises fewer hexoses than n but is otherwise identical to n may contribute a fraction of its actual abundance to a_n. If p is glycated g times, $\mathrm{Hex}(p) + g$ will equal Hex(n), which makes p and n indistinguishable and transfers the fraction $c_g x_p$ of p's actual abundance x_p to a_n (c_g is the overall abundance of proteoforms with g glucoses attached due to glycation). For example, single glycation of the glycoform A2G0F/A2G1F (7 Hex, 8 HexNAc, 2 Fuc) yields a proteoform whose monosaccharide composition equals the one of A2G0F/A2G2F. Hence, part of the abundance observed for the “8 Hex, 8 HexNAc, 2 Fuc” peak results from singly-glycated A2G0F/A2G1F.
3. n will contribute a fraction of its actual abundance to the observed abundance of any monosaccharide composition s which comprises more hexoses than n but is otherwise identical to n. If n is glycated g times, $\mathrm{Hex}(n) + g$ will equal Hex(s), which makes n and s indistinguishable and transfers the fraction $c_g x_n$ of n's actual abundance x_n to a_s (i.e., the abundance observed for s). For instance, a single glycation of the glycoform A2G0F/A2G2F yields a proteoform whose monosaccharide composition equals the one of A2G1F/A2G2F (9 Hex, 8 HexNAc, 2 Fuc). Hence, part of the actual abundance of the “8 Hex, 8 HexNAc, 2 Fuc” peak is lost to the A2G1F/A2G2F peak.

To systematically gather all possible transfers of abundance between proteoforms, we constructed a graph $G = (V, E)$, comprising the sets V and E of nodes and edges, respectively (see Figure 3 in manuscript). V contained a node n for each monosaccharide composition. Each node $n \in V$ was associated with an observed abundance a_n as well as an (initially unknown) actual abundance x_n. E contained a weighted directed edge e_{nm} for each pair n, m of nodes whose monosaccharide compositions differed by at least one hexose, but were identical otherwise. Hence, each edge represented glycation-related addition of glucose moieties. For example, the edge connecting $n =$ “6 Hex, 8 HexNAc, 2 Fuc” and $m =$ “7 Hex, 8 HexNAc, 2 Fuc” described a single glycation in glycoform A2G0F/A2G0F, which yields a proteoform whose monosaccharide composition resembles the one of A2G0F/A2G1F. Furthermore, the weight c_{nm} associated with e_{nm} corresponded to the overall abundance of protein species with $\mathrm{Hex}(m) - \mathrm{Hex}(n)$ glycations. In the example above, c_{nm} was the abundance of singly-glycated species as measured in the PNGase F-treated samples.

The glycation graph proposed the following formula which describes the three factors that influence the observed abundance of each monosaccharide composition:

$$a_n = x_n + \sum_{p \in \mathrm{Pred}(n)} x_p c_{pn} - \sum_{s \in \mathrm{Succ}(n)} x_n c_{ns} \tag{1}$$

for all $n \in V$, where $\mathrm{Pred}(n)$ and $\mathrm{Succ}(n)$ denote the sets of predecessors and successors of node n, respectively. If we solve equation (1) for x_n, we obtain

$$x_n = \frac{a_n - \sum_{p \in \mathrm{Pred}(n)} x_p c_{pn}}{1 - \sum_{s \in \mathrm{Succ}(n)} c_{ns}} , \tag{2}$$

which shows that the actual abundance of any monosaccharide composition (node) n may be determined once the actual abundances of all of its predecessors have been calculated. Since the glycation graph is directed and acyclic, the following simple algorithm allows to calculate all actual abundances: (a) Sort the graph topologically. (b) Calculate x_n according to equation (2), starting with the first node and proceeding according to the topological sort. Thereby, the algorithm ensures that, whenever it calculates x_n for any n, the true abundances x_p of all predecessors $p \in \mathrm{Pred}(n)$ are already known.

Correction algorithm: Implementation

The algorithm was implemented in Python v3.5 (Python Software Foundation, `https://www.python.org`), importing NumPy v1.15.4[1] and pandas v0.20.3[2] from the SciPy stack, as well as the modules multiset v2.0.3 (`https://github.com/wheerd/multiset`), NetworkX v2.1[3], and uncertainties v3.0.2 (Eric O. Lebigot, `https://pythonhosted.org/uncertainties`). The graphical user interface was built with the Qt 5 framework bound via PyQt5 v5.10.1 and PyQtChart v5.10.1 (Riverbank Computing, `https://www.riverbankcomputing.com`).

In the remainder of this document, we will refer to the algorithm as "CAFOG" ("Correct Abundances FOr Glycation bias").

Materials

Avastin® (Roche, lot B7214H09 expiring 03/2018) and Prolia® (Amgen, "old" lot 1069106B expiring 06/2016 and "new" lot EU 1092617B expiring 09/2018) were bought from a local pharmacy. NISTmAb (National Institute of Standards and Technology monoclonal antibody reference material 8671, lot 14HB-D-001) was obtained from the National Institute of Standards and Technology.

Identification and relative quantification of mAb glycoforms

mAb glycosylation variants were identified and relatively quantified as described by Regl *et al.*[4]. In brief, samples were centrifuged and diluted before separation on a monolithic PS-DVB column (ProSwift™ RP-10R, 50 × 1.0 mm i.d.; Thermo Scientific™, Sunnyvale, CA, USA) using a 15 min acetonitrile gradient, and detection on a quadrupole-Orbitrap

mass spectrometer (Thermo Scientific™ Q Exactive™, Bremen, Germany). Glycoforms were identified based on the molecular mass using the BioPharma Finder software (Thermo Scientific™). Extracted ion current chromatograms (XICCs) derived from theoretical masses of the identified mAb glycoforms were generated in order to obtain fractional abundances. Denosumab samples were additionally treated with Carboxypeptidase B from pig pancreas (Roche, Mannheim, Germany) to remove *C*-terminal lysine residues using a substrate to enzyme ratio of 25 : 1 (w/w) for 30 min at 37 °C before analysis.

Released *N*-glycan analysis

N-glycans were released using PNGase F (Roche, Mannheim, Germany), separated by ultracentrifugal filtration and concentrated by centrifugal evaporation. Released *N*-glycans were labeled with an excess of 2-aminobenzamide (2-AB, Sigma-Aldrich, Steinheim, Germany) followed by gel filtration using Sephadex G10 (GE Healthcare, Little Chalfont, UK) to remove free label. Hydrophilic interaction chromatography (HILIC) using an Acquity BEH Glycan (2.1×100 mm column; Waters Corporation, Milford, MA, USA) was employed to separate 2-AB-labeled glycans. Fluorescence detection (FLD) was performed at an excitation wavelength of 250 nm and an emission wavelength of 428 mm.

Forced glycation

10 mg mL^{-1} NISTmAb (National Institute of Standards and Technology monoclonal antibody reference material 8671, lot 14HB-D-001) in 12.5 mM *L*-histidine, 12.5 mM *L*-histidine HCl (*pH* 6.0) was mixed with an equal volume of 1 M glucose, yielding a final glucose concentration of 500 mM. The mixture was incubated in the dark at 40 °C while shaking (1000 rpm). Samples were drawn at ten time points (0, 0.25, 0.5, 1, 2, 4, 6, 8, 24, and 28 h) and stored at –80 °C.

Stressed samples were rebuffered to 175 mM ammonium acetate (Merck) five times using Sartorius Vivaspin 500 MWCO 30 kDa centrifugal concentrators (Sigma-Aldrich) at 14000 g and 4 °C. Subsequently, the protein concentration was measured employing a nanophotometer (P330, Implen GmbH, Munich, Germany) at 280 nm, assuming an extinction coefficient of 211480 L mol^{-1} cm^{-1}. Half of each sample was digested with carboxypeptidase B (Roche, 1 h, 37 °C, mAb to enzyme ratio 5 : 1 w/w); the other half was digested with both carboxypeptidase B and non-reducing Rapid™ PNGase F (New England BioLabs, 5 min, 75 °C, 1 µL PNGase F per 20 µg mAb). Following digestion, samples were again rebuffered to 175 mM ammonium acetate and diluted to a final concentration of 0.2 mg mL^{-1}.

Mass spectrometry was conducted employing static nano-electrospray ionization on a Thermo Scientific™ Q Exactive™ Plus benchtop quadrupole-Orbitrap mass spectrometer equipped with a Nanospray Flex™ ion source with borosilicate emitters (all from Thermo Fisher Scientific). The instrument settings were as follows: 1.4 kV spray voltage, 250 °C capillary temperature, 100.0 S-lens RF level, 60 eV in-source collision induced dissociation, scan range of 200 to 8000 m/z in high mass range mode at a resolution setting of 35000 at 200 m/z, 100 ms maximum injection time with an automatic gain control target of

5×10^6. Spectra were acquired using rolling averaging for 500 scans.

Glycated species (0 to 7 hexoses) and glycoforms were quantified by integrating peaks at the respective m/z values in the raw mass spectra, including peaks in charge states $z = +24$ to $+27$ for glycation and $z = +24$ to $+30$ for glycoforms, respectively. Integration was done *via* a custom R script, available with the raw data (see below). Prior to integration, mass spectrometry data files were converted from RAW to mzML format using ThermoRawFileParser v1.1.8[5].

Code and data availability

CAFOG source code and documentation are freely available from GitHub (`https://github.com/cdl-biosimilars/cafog`) and in Supplementary File 1. All input files and data analysis scripts used in this study are available as ZIP archive (Supplementary File 2).

Raw data for bevacizumab has been previously published[4] and is available from the ProteomeXchange Consortium *via* the PRIDE partner repository[6] with the dataset identifier PXD011017. Raw data for denosumab and NISTmAb is available from Zenodo (`https://doi.org/10.5281/zenodo.3631072`).

Supplementary figures

Supplementary figure 1

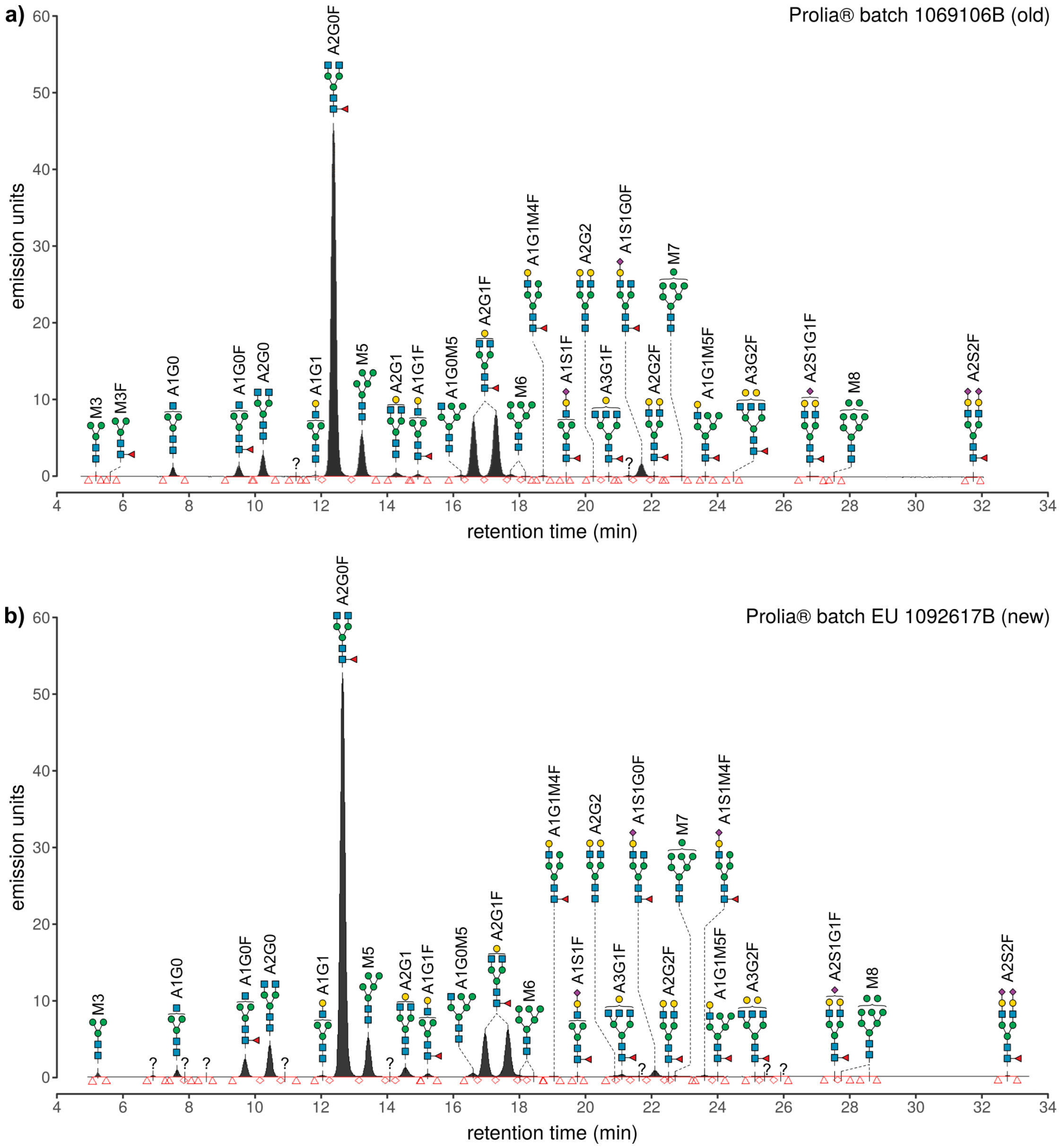

HILIC-FLD analysis of 2-aminobenzamide-labeled *N*-glycans enzymatically released from two batches of denosumab (a and b). Red empty triangles and diamonds below the baseline denote peak integration boundaries used for relative quantification. Question marks indicate signals that could not be annotated.

Supplementary tables

Supplementary table 1

N-glycan structures and monosaccharide compositions. All structures appear in Symbol Nomenclature For Glycans (SNFG) representation[7]. Glycoform names follow the Zhang nomenclature[8].

Name	Structure	Hex	HexNAc	Fuc	Neu5Ac
A1G0	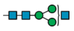	3	3	0	0
A1G0F	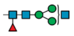	3	3	1	0
A1G0M5	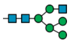	5	3	0	0
A1G1	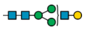	4	3	0	0
A1G1F	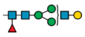	4	3	1	0
A1G1M4F	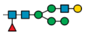	5	3	1	0
A1G1M5F	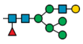	6	3	1	0
A1S1F	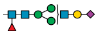	4	3	1	1
A1S1M4F	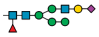	5	3	1	1
A2G0	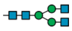	3	4	0	0
A2G0F	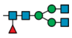	3	4	1	0
A2G1	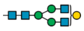	4	4	0	0
A2G1F	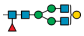	4	4	1	0
A2G2	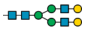	5	4	0	0
A2G2F	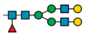	5	4	1	0
A2S1G0F	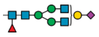	4	4	1	1
A2S1G1F	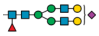	5	4	1	1
A2S2F	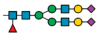	5	4	1	2
A3G0	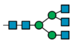	3	5	0	0
A3G0F	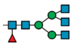	3	5	1	0
A3G1F	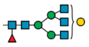	4	5	1	0
A3G2F	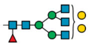	5	5	1	0
GnF	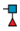	0	1	1	0
M3	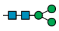	3	2	0	0
M3F	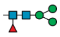	3	2	1	0
M5	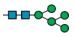	5	2	0	0
M6	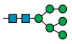	6	2	0	0
M7	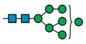	7	2	0	0
M8	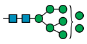	8	2	0	0

Supplementary files

Supplementary file 1: `cafog_source_code.zip`

A ZIP archive containing the source code of CAFOG. To run the program in source or create a standalone executable, follow the instructions in `README.md`.

Supplementary file 2: `data.zip`

Files in this ZIP archive allow to reproduce all results presented in the manuscript. CSV files comply with RFC 4180[9], with a hash character in column 1 denoting comment lines.

The following folders contain CAFOG input files and results:

- `bevacizumab/` – data on four fermentation samples of a bevacizumab biosimilar (`day5/`, `day10/`, `day14/`, and `capel/`) as well as the originator Avastin® (`ref/`)
- `denosumab/` – data on two production batches of Prolia® (`old/` and `new/`)
- `nist_mab/` – data on a forced glycation experiment with NISTmAb (ten subfolders, `0h/` to `28h/`)

Each of these folders contains the files

- `glycan_library.csv` – the glycan library
- `glycation.csv` – glycation counts with abundances and errors
- `glycoforms.csv` – glycoforms with abundances and errors
- `results.csv` – CAFOG results as obtained by executing the shell command

```
python cafog.py \
  -f glycoforms.csv \
  -g glycation.csv \
  -l glycan_library.csv \
  > results.csv
```

Files named `mass_spectrum_glycation.csv` and `mass_spectrum_intact.csv` contain raw mass spectra (i.e., m/z and intensity values). Such files are available in the folders

- `bevacizumab/day5/` – used for Figures 1b and a
- `denosumab/new/` – used for the purple lines in Figures 6b and a
- `denosumab/old/` – used for the orange lines in Figures 6b and a
- `nist_mab/0h` – used for Figures 5a and b
- `nist_mab/28h` – used for Figures 5c and d

The file `denosumab/released_glycans.csv` describes released glycan abundances for the denosumab batches.

In addition, `supporting_information.Rmd` contains this document in R Markdown format[10]. Conversion to PDF via `knitr`[11] requires `angew.csl`, `orcid.pdf`, `references.bib`, `si_template.tex`, and all files in `glycan_structures/` and `supplementary_figures/`.

Code for data analysis and figures

Charts were created in R[12] using packages from the tidyverse[13]. Publication-quality figures were prepared with Inkscape v0.91 (`https://inkscape.org`) and GIMP v2.8.16 (`https://www.gimp.org`).

```
library(tidyverse)
library(broom)
library(fs)
```

Use colorblind safe red/blue color scheme from ColorBrewer[14] for observed and actual abundances.

```
RdBu <- c("#ca0020", "#0571b0")
```

A vector for mapping monosaccharide compositions (Fuc/Hex/HexNAc/Neu5Ac) to glycoforms.

```
glycoform_names <- c(
  "0/10/4/0" = "M5•M5",
  "1/3/4/0" = "A2G0F",
  "1/6/8/0" = "A2G0•A2G0F",
  "1/8/6/0" = "A2G0F•M5",
  "2/6/6/0" = "A1G0F•A1G0F",
  "2/6/7/0" = "A2G0F•A1G0F",
  "2/6/8/0" = "A2G0F•A2G0F",
  "2/7/7/0" = "A1G0F•A2G1F",
  "2/7/8/0" = "A2G0F•A2G1F",
  "2/7/8/1" = "A2G0F•A2S1G0F",
  "2/8/7/0" = "A2G1F•A1G1F",
  "2/8/8/0" = "A2G1F•A2G1F",
  "2/8/8/1" = "A2G0F•A2S1G1F",
  "2/9/8/0" = "A2G1F•A2G2F",
  "2/9/8/1" = "A2G1F•A2S1G1F",
  "2/10/8/0" = "A2G2F•A2G2F",
  "other" = "other glycoforms"
)
```

Save generated figures in `./raw_figures/`. Create this folder if necessary.

```
dir.create(file.path(".", "raw_figures"), showWarnings = FALSE)
ggsave_default <- function(filename, ...) {
  ggsave(str_glue("raw_figures/{filename}.svg"), units = "cm", ...)
}
```

Figure 1: Glycosylation and glycation

a) Mass spectrum of intact bevacizumab

`load_mass_spectrum` loads a mass spectrum stored in the CSV file `filename` (with column names `mz` and `intensity`) into a data frame with three columns: `mz`, `abundance` (relative abundance), and `batch`.

```
load_mass_spectrum <- function(filename, batch = NULL) {
  batch <- batch %||% filename
  suppressMessages(read_csv(filename, comment = "#")) %>%
    mutate(
      abundance = intensity / max(intensity) * 100,
      batch = batch
    ) %>%
    select(-intensity)
}
```

Load a mass spectrum of intact bevacizumab (sample from day 5).

```
df <- load_mass_spectrum("bevacizumab/day5/mass_spectrum_intact.csv")
```

Create a line chart (abundance vs. m/z) of the most abundant charge state (39+).

```
ggplot(df) +
  geom_line(aes(mz, abundance), color = "#aa4400") +
  scale_x_continuous(
    name = "m/z",
    limits = c(3770, 3870),
    breaks = c(3800, 3850)
  ) +
  scale_y_continuous(
    name = "relative abundance (%)",
    breaks = c(0, 50, 100),
    expand = c(0, 0)
  ) +
  theme_bw() +
  theme(
    axis.line.y.left = element_line(),
    panel.border = element_blank(),
    panel.grid = element_blank()
  )
```

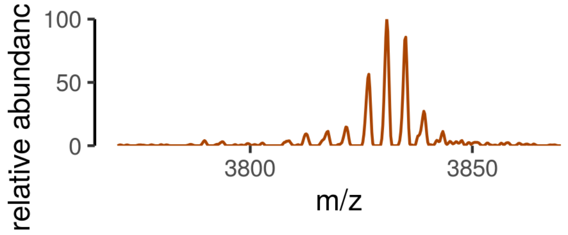


```
ggsave_default("figure1a", width = 8, height = 3)
```

b) Mass spectrum of PNGase F-digested bevacizumab

Load a mass spectrum of PNGase F-digested bevacizumab (sample from day 5).

```
df <- load_mass_spectrum("bevacizumab/day5/mass_spectrum_glycation.csv")
```

Create a line chart (abundance vs. m/z) of the most abundant charge state (45+).

```
ggplot(df) +
  geom_line(aes(mz, abundance), color = "#aa4400") +
  scale_x_continuous(
    name = "m/z",
    limits = c(3245, 3275)
  ) +
  scale_y_continuous(
    name = "relative abundance (%)",
    breaks = c(0, 50, 100),
    expand = c(0, 0)
  ) +
  theme_bw() +
  theme(
    axis.line.y.left = element_line(),
    panel.border = element_blank(),
    panel.grid = element_blank()
  )
```

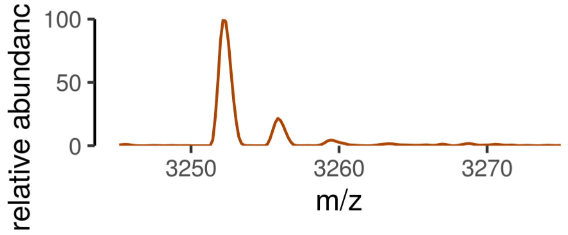

```
ggsave_default("figure1b", width = 8, height = 3)
```

Figure 2: Observed vs. actual abundance

This figure was drawn in Inkscape.

Figure 3: Glycation graph

This figure was prepared with Gephi v0.9.2 (`https://gephi.org`), followed by manual fine-tuning with Inkscape.

Figure 4: Quantification of bevacizumab glycoforms

`load_cafog_results` loads CAFOG results stored in the CSV file `filename` into a data frame with the following columns:

- `composition` (character) – monosaccharide composition (Fuc/Hex/HexNAc/Neu5Ac)
- `type` (character) – actual or observed
- `batch` (character) – the `batch` as specified in the formal arguments
- `abundance` (double) – relative abundance
- `error` (double) – measurement error

```
load_cafog_results <- function(filename, batch) {
  read_csv(filename) %>%
    replace_na(list(Fuc = 0, Hex = 0, HexNAc = 0, Neu5Ac = 0)) %>%
    unite(Fuc:Neu5Ac, col = "composition", sep = "/") %>%
    arrange(desc(abundance)) %>%
    rename(observed = abundance, actual = corr_abundance) %>%
    pivot_longer(
      c(observed, actual),
      names_to = "type",
      values_to = "abundance"
    ) %>%
    mutate(
      type = as_factor(type),
      error = case_when(
        type == "observed" ~ abundance_error,
        type == "actual"   ~ corr_abundance_error
      ),
      batch = batch
    ) %>%
    select(composition, type, batch, abundance, error)
}
```

`load_batches` loads CAFOG results for several batches, which are stored in the files `./protein/.../results.csv`. The function returns a list of data frames as generated by `load_cafog_results`.

```
load_batches <- function(protein, ...) {
  map(
    list(...),
    function(batch) {
      file.path(protein, batch, "results.csv") %>%
        load_cafog_results(batch)
    }
  )
}
```

`aggregate_glycoforms` only keeps `n_bars` − 1 glycoforms that have the highest observed abundance in a dataset `df`, aggregating the lower abundant ones into an observation called `other glycoforms`.

```
aggregate_glycoforms <- function(df, n_bars) {
  top_compositions <-
    df %>%
    filter(type == "observed") %>%
    group_by(composition) %>%
    summarize(total_abundance = sum(abundance)) %>%
    mutate(rank = min_rank(desc(total_abundance))) %>%
    filter(rank <= n_bars - 1) %>%
    select(-total_abundance)

  top_bars <-
    df %>%
    inner_join(top_compositions, by = "composition")

  df %>%
    anti_join(top_bars, by = c("composition", "type", "batch")) %>%
    group_by(type, batch) %>%
    summarise_if(is.numeric, sum) %>%
    mutate(composition = "other glycoforms", rank = n_bars) %>%
    bind_rows(top_bars) %>%
    ungroup()
}
```

Load CAFOG results for five bevacizumab samples and aggregate all but the seven most abundant glycoforms.

```
df <-
  "bevacizumab" %>%
  load_batches("day5", "day10", "day14", "capel", "ref") %>%
  bind_rows() %>%
  aggregate_glycoforms(n_bars = 8) %>%
  mutate(
    batch =
      batch %>%
      fct_relevel("day5", "day10", "day14", "capel", "ref") %>%
      fct_recode(
        "day 5" = "day5",
        "day 10" = "day10",
        "day 14" = "day14",
        "capture eluate" = "capel",
        "reference product" = "ref"),
    type = type %>% fct_rev()
  )
```

Draw one bar chart for each batch and arrange the charts in a single row.

```
df %>%
  mutate(
    composition = composition %>%
      recode(!!!glycoform_names) %>%
      factor() %>%
      fct_reorder(rank) %>%
      fct_rev()
  ) %>%
  ggplot(aes(composition)) +
  geom_col(
    aes(y = abundance, fill = type),
    position = position_dodge(.9),
    alpha = .5,
    show.legend = FALSE
  ) +
  geom_errorbar(
    aes(
      ymin = abundance - error,
      ymax = abundance + error,
      group = type
    ),
    position = position_dodge(.9),
    width = .5,
    size = .25
```

```
  ) +
  geom_hline(yintercept = 0, size = .35) +
  scale_x_discrete(name = NULL) +
  scale_y_continuous(name = "fractional abundance (%)", expand = c(0, 0)) +
  scale_fill_manual(values = rev(RdBu)) +
  coord_flip() +
  facet_wrap(vars(batch), nrow = 1) +
  theme_bw() +
  theme(
    axis.ticks.y = element_blank(),
    panel.border = element_blank(),
    panel.grid.major.y = element_blank(),
    panel.grid.minor = element_blank(),
    plot.margin = margin(.5, .5, .5, .5, "mm"),
    strip.background = element_blank(),
    strip.placement = "outside",
    strip.text = element_text(face = "bold")
  )
```

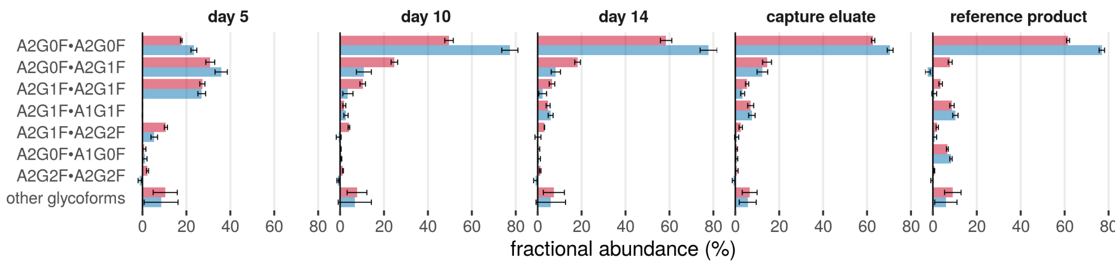


```
ggsave_default("figure4", width = 21, height = 5)
```

Figure 5: Forced glycation of NISTmAb

a–d) Mass spectra

Load mass spectra of intact and PNGase F-digested NISTmAb before and after 28 h of forced glycation.

```
nistmab_spectra <-
  c(
    "nist_mab/0h/mass_spectrum_intact.csv",
    "nist_mab/0h/mass_spectrum_glycation.csv",
    "nist_mab/28h/mass_spectrum_intact.csv",
    "nist_mab/28h/mass_spectrum_glycation.csv"
```

```
  ) %>%
  map_dfr(load_mass_spectrum) %>%
  extract(
    batch,
    into = c("time", "type"),
    regex = "(\\d+)h/mass_spectrum_([:lower:]+)\\."
  )
```

`draw_forced_glycation_spectra` is a wrapper for drawing the subcharts, since all of them should look the same.

```
draw_forced_glycation_spectra <- function(type, lower_mz, upper_mz) {
  nistmab_spectra %>%
    filter(
      type == {{type}},
      mz %>% between(lower_mz, upper_mz)
    ) %>%
    group_by(time) %>%
    mutate(abundance = abundance / max(abundance) * 100) %>%
    ggplot(aes(mz, abundance)) +
    geom_area(aes(fill = factor(time)), show.legend = FALSE) +
    geom_hline(yintercept = 0, size = .35) +
    scale_x_continuous(name = "m/z") +
    scale_y_continuous(
      name = "relative abundance (%)",
      expand = expansion(add = 0)
    ) +
    scale_fill_manual(values = c("#9970ab", "#5aae61")) +
    facet_wrap(vars(time), ncol = 1) +
    theme_bw() +
    theme(
      panel.border = element_blank(),
      axis.line.y.left = element_line(),
      panel.grid = element_blank(),
      strip.background = element_blank()
    )
}
```

Draw area charts of abundance vs m/z for intact NISTmAb (charge state 27+) …

```
draw_forced_glycation_spectra("intact", 5450, 5550)
```

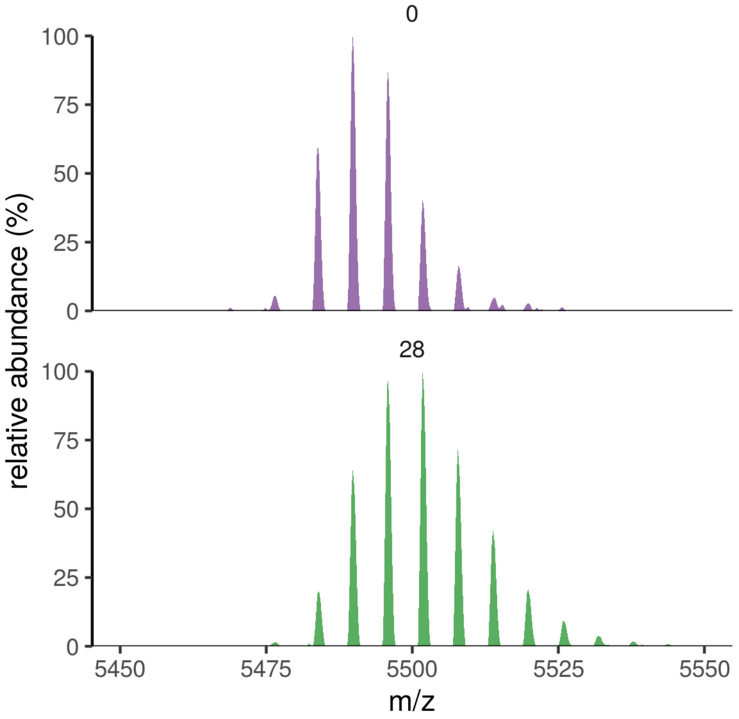


```
ggsave_default("figure5ac", width = 10, height = 10)
```

… and for PNGase F-treated protein (charge state 25+).

```
draw_forced_glycation_spectra("glycation", 5800, 5900)
```

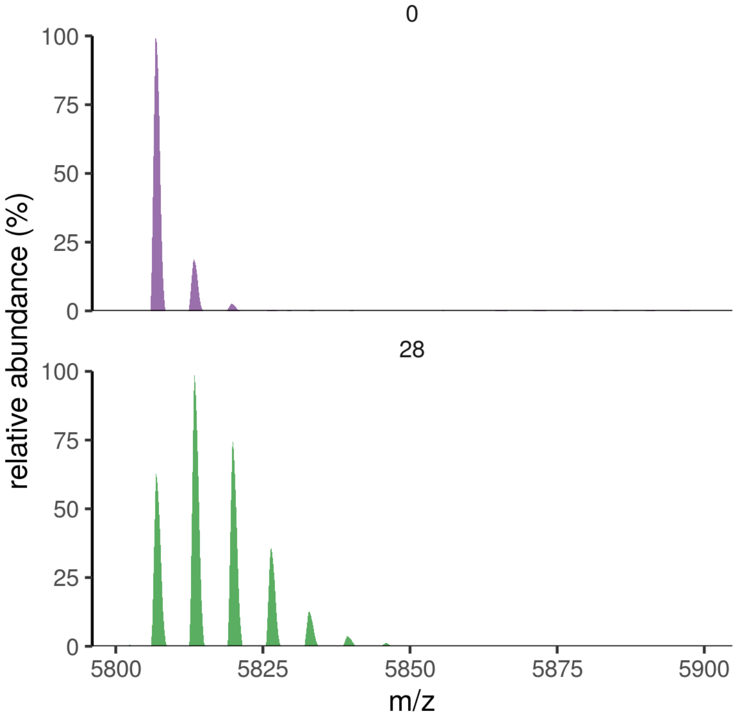


```
ggsave_default("figure5bd", width = 10, height = 10)
```

e) Glycation abundance

Load glycation abundances and draw a line chart.

```
dir_ls("nist_mab", regexp = "/glycation\\.csv", recurse = TRUE) %>%
  map_dfr(
    read_csv,
    .id = "file",
    col_names = c("hex", "abundance", "error"),
    comment = "#"
  ) %>%
  extract(
    file,
    into = "time",
    regex = "([\\d\\.]+)",
    convert = TRUE
  ) %>%
  filter(hex != 7) %>%
  ggplot(aes(time, abundance, color = factor(hex))) +
```

```
  geom_line(show.legend = FALSE) +
  geom_point(alpha = .25, show.legend = FALSE) +
   geom_errorbar(
    aes(ymin = abundance - error, ymax = abundance + error),
    width = .5,
    size = .5,
    show.legend = FALSE
  ) +
  scale_x_continuous(
    name = "time (h)",
    breaks = c(0, 28),
    limits = c(0, 30)
  ) +
  scale_y_continuous(name = "fractional abundance (%)") +
  scale_color_viridis_d(name = "glycations", option = "C", end = 6/7) +
  theme_bw() +
  theme(panel.grid = element_blank()) +
  NULL
```

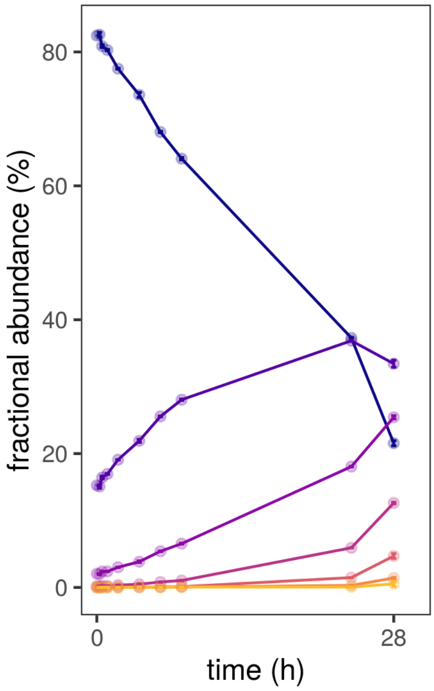


```
ggsave_default("figure5e", width = 6, height = 9.4)
```

f–g) Glycoform abundance correction

The following table lists all glycoforms that were quantified, as well as their monosaccharide composition (h, Hex; n, HexNAc; f, Fuc).

```
nistmab_glycoforms <- tribble(
  ~h, ~n, ~f, ~name,
   6,  8,  2, "A2G0F/A2G0F",
   7,  8,  2, "A2G0F/A2G1F",
   8,  8,  2, "A2G1F/A2G1F",
   9,  8,  2, "A2G1F/A2G2F",
  10,  8,  2, "A2G2F/A2G2F",
  11,  8,  2, "A2G2F/A2G2F+1Hex",
  12,  8,  2, "A2G2F/A2G2F+2Hex",
  13,  8,  2, "A2G2F/A2G2F+3Hex",
  14,  8,  2, "A2G2F/A2G2F+4Hex",
  15,  8,  2, "A2G2F/A2G2F+5Hex",
   6,  7,  2, "A1G0F/A2G0F",
   3,  4,  1, "un/A2G0F",
   4,  4,  1, "un/A2G1F",
   5,  4,  1, "un/A2G2F",
   6,  4,  1, "un/A2G2F+1Hex",
   7,  4,  1, "un/A2G2F+2Hex",
)
```

Load CAFOG results.

```
nistmab_results <-
  dir_ls("nist_mab", regexp = "results\\.csv", recurse = TRUE) %>%
  map_dfr(
    ~read_csv(.x) %>%
      left_join(
        nistmab_glycoforms,
        by = c(Hex = "h", HexNAc = "n", Fuc = "f")
      ) %>%
      select(name, abundance:corr_abundance_error),
    .id = "file"
  ) %>%
  extract(file, into = "time", regex = "/([\\d\\.]+)h", convert = TRUE)
```

Accumulate abundances for all glycoforms not listed in the table `nistmab_glycoforms` (these glycoforms were automatically generated by CAFOG, since the algorithm generates combinations of all available glycans).

```
nistmab_abundances <-
  nistmab_results %>%
  filter(!is.na(name)) %>%
  bind_rows(
    nistmab_results %>%
      filter(is.na(name)) %>%
      group_by(time) %>%
      summarise(
        abundance = sum(abundance),
        abundance_error = sqrt(sum(abundance_error^2)),
        corr_abundance = sum(corr_abundance),
        corr_abundance_error = sqrt(sum(corr_abundance_error^2)),
      ) %>%
      mutate(name = "other glycoforms")
  )
```

Determine the seven most abundant glycoforms in the untreated sample.

```
nistmab_top7_glycoforms <-
  nistmab_abundances %>%
  filter(time == 0) %>%
  arrange(desc(abundance)) %>%
  head(7) %>%
  pull(name)
```

Accumulate the abundance of all other glycoforms as “other glycoforms”.

```
nistmab_top7_abundances <-
  nistmab_abundances %>%
  mutate(
    name = case_when(
      name %in% nistmab_top7_glycoforms ~ name,
      TRUE                              ~ "other glycoforms"
    )
  ) %>%
  group_by(time, name) %>%
  summarise(
    abundance = sum(abundance),
    abundance_error = sqrt(sum(abundance_error^2)),
    corr_abundance = sum(corr_abundance),
    corr_abundance_error = sqrt(sum(corr_abundance_error^2)),
  ) %>%
  pivot_longer_spec(
    tribble(
```

```
      ~.name,                 ~.value,     ~type,
      "abundance",            "abundance", "observed",
      "abundance_error",      "error",     "observed",
      "corr_abundance",       "abundance", "actual",
      "corr_abundance_error", "error",     "actual"
    )
  ) %>%
  mutate(
    name = name %>%
      str_replace("/", "•") %>%
      factor() %>%
      fct_reorder(abundance) %>%
      fct_rev(),
    type = type %>%
      factor() %>%
      fct_rev()
  ) %>%
  ungroup()
```

Draw a line chart that depicts the change of abundances for the selected glycoforms with time during forced glycation. One chart shows observed abundances, the other one depicts the corrected values.

```
nistmab_top7_abundances %>%
  ggplot(aes(time, abundance, color = name)) +
  geom_line() +
  geom_point(alpha = .25) +
  geom_errorbar(
    aes(ymin = abundance - error, ymax = abundance + error),
    width = .5,
    size = .5
  ) +
  scale_x_continuous(name = "time (h)") +
  scale_y_continuous(name = "fractional abundance (%)") +
  scale_color_viridis_d(name = "glycoform", option = "D") +
  facet_wrap(vars(type)) +
  theme_bw() +
  theme(
    strip.background = element_blank(),
    strip.text = element_text(face = "bold")
  )
```

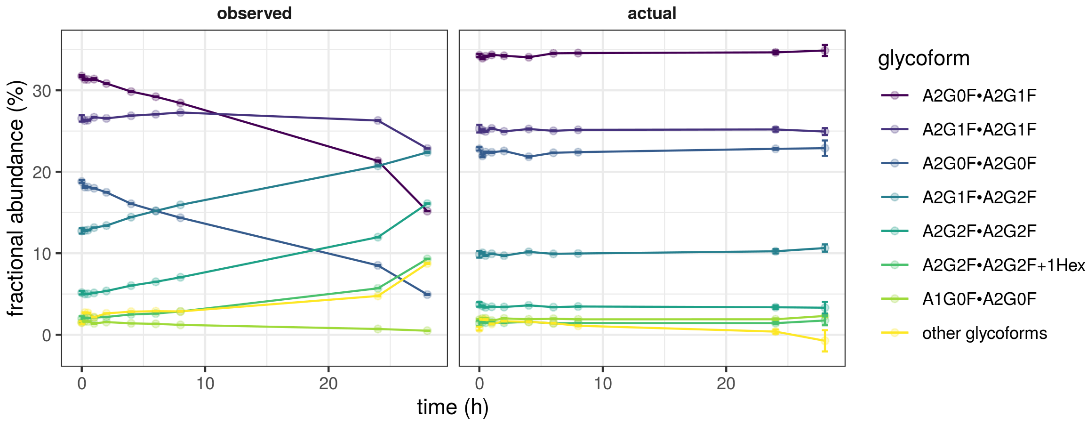


```
ggsave_default("figure5fg", width = 20, height = 8)
```

Perform a linear regression for each glycoform (actual abundance depending on time) and report the corresponding slopes and significances.

```
nistmab_top7_abundances %>%
  filter(type == "actual") %>%
  split(.$name) %>%
  map(~lm(abundance ~ time, data = .)) %>%
  map_dfr(tidy, .id = "name") %>%
  filter(term == "time") %>%
  select(glycoform = name, slope = estimate, p = p.value) %>%
  mutate(`< 0.05` = case_when(p < 0.05 ~ "*", TRUE ~ "")) %>%
  knitr::kable(digits = 2)
```

glycoform	slope	p	$<$ 0.05
A2G0F • A2G1F	0.02	0.00	*
A2G1F • A2G1F	0.00	0.50	
A2G0F • A2G0F	0.02	0.08	
A2G1F • A2G2F	0.02	0.00	*
A2G2F • A2G2F	-0.01	0.12	
A2G2F • A2G2F+1Hex	0.00	0.18	
A1G0F • A2G0F	0.01	0.10	
other glycoforms	-0.07	0.00	*

Figure 6: Hexosylation bias in denosumab

a) Mass spectra of the intact protein

Define several helper functions for aligning the charge states: `mz_to_mass` converts a vector of m/z values to a vector of zero-charge masses, given the charge `z` and the mass of the charge agent (default: the average mass of a proton as estimated from its isotopic abundance in organic meterials[15]).

```
mz_to_mass <- function(mz, z, charge_agent_mass = 1.007968) {
  (mz - charge_agent_mass) * z
}
```

Conversely, `mz_to_mass` converts a vector of masses to a vector of m/z values.

```
mass_to_mz <- function(mass, z, charge_agent_mass = 1.007968) {
  mass / z + charge_agent_mass
}
```

`deconvolute` allows comparison of several charge states: It converts m/z values in a `spectrum` to zero-charge masses, once for each supplied value in the vector `charge_states`, and then discards masses outside of the interval [`lower`, `upper`].

```
deconvolute <- function(spectrum, charge_states, lower, upper) {
  charge_states %>%
    map_dfr(
      function(z)
        spectrum %>% mutate(mass = mz_to_mass(mz, z), z = z)
    ) %>%
    filter(between(mass, lower, upper))
}
```

Load mass spectra of both batches of intact denosumab and calculate zero-charge masses for charge state 55+.

```
intact_spectrum_old <-
  load_mass_spectrum("denosumab/old/mass_spectrum_intact.csv", "old")

intact_spectrum_new <-
  load_mass_spectrum("denosumab/new/mass_spectrum_intact.csv", "new")

intact_spectra <-
  bind_rows(intact_spectrum_old, intact_spectrum_new) %>%
  mutate(batch = as_factor(batch))
```

```r
intact_zero_charge_spectra <-
  intact_spectra %>%
  deconvolute(55L, 146500, 148500)
```

Draw a line chart (abundance vs. m/z) including a secondary x-axis with the zero-charge mass corresponding to the m/z values.

```r
ggplot(intact_zero_charge_spectra, aes(mz, abundance)) +
  geom_area(
    aes(fill = batch),
    show.legend = FALSE
  ) +
  geom_line(
    aes(color = batch),
    show.legend = TRUE,
    size = .25
  ) +
  annotate(
    "text",
    x = mass_to_mz(146700, 55),
    y = 87.5,
    label = "55+",
    fontface = "bold"
  ) +
  scale_color_manual(values = c("black", "#ff6600")) +
  scale_fill_manual(values = c("gray95", NA)) +
  scale_x_continuous(
    name = "m/z",
    sec.axis = sec_axis(
      ~mz_to_mass(., 55) / 1000,
      name = "mass (kDa)",
      breaks = c(146.5, 147.5, 148.5)
    )
  ) +
  scale_y_continuous(
    name = "relative abundance (%)",
    breaks = c(0, 50, 100),
    limits = c(-1, 100),
    expand = c(0, 0)
  ) +
  theme_bw() +
  theme(
    axis.line.x.top = element_line(),
    axis.line.y.left = element_line(),
```

```
    legend.background = element_blank(),
    legend.position = c(.8, .5),
    panel.border = element_blank(),
    panel.grid = element_blank(),
    plot.margin = margin(1.5, .1, .1, .1, "mm"),
    strip.text = element_blank()
  )
```

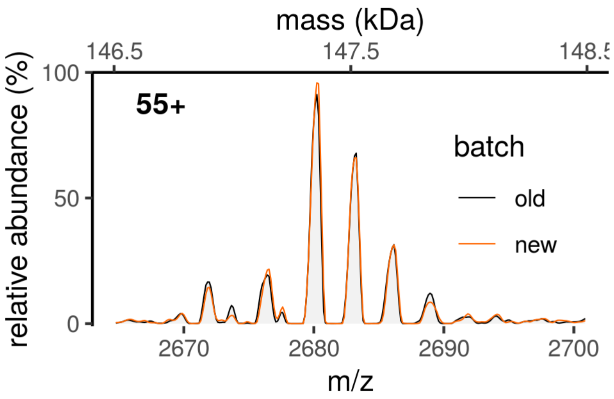


```
ggsave_default("figure6a", width = 8, height = 5.4)
```

b) Glycoform quantification

Load CAFOG results for two denosumab batches and aggregate all but the seven glycoforms that are most abundant on the dataset level.

```
df_mab2 <-
  "denosumab" %>%
  load_batches("old", "new") %>%
  bind_rows()

df_mab2_agg <-
  df_mab2 %>%
  aggregate_glycoforms(n_bars = 8) %>%
  mutate(
    composition = composition %>%
      recode(!!!glycoform_names) %>%
      as_factor() %>%
      fct_reorder(rank)
  )
```

Draw one bar chart for each batch and arrange the charts side-by-side by faceting (the y-axes share a common scale).

```r
ggplot(df_mab2_agg, aes(composition)) +
  geom_col(
    aes(y = abundance, fill = batch),
    position = position_dodge(.9),
    alpha = .4,
    show.legend = FALSE
  ) +
  geom_errorbar(
    aes(
      ymin = abundance - error,
      ymax = abundance + error,
      group = batch
    ),
    position = position_dodge(.9),
    width = .5,
    size = .25
  ) +
  scale_x_discrete(
    name = NULL,
    limits = rev(levels(df_mab2_agg$composition)),
    expand = c(0, 0)
  ) +
  scale_y_continuous(
    name = "fractional abundance (%)",
    expand = c(0, 0),
    position = "right"
  ) +
  scale_fill_manual(values = c("#ff6600", "black")) +
  coord_flip() +
  facet_wrap(vars(type)) +
  theme_bw() +
  theme(
    axis.line.y = element_line(),
    axis.ticks = element_blank(),
    panel.border = element_blank(),
    panel.grid.major.y = element_blank(),
    panel.grid.minor = element_blank(),
    plot.margin = margin(.1, .1, .1, .1, "mm"),
    strip.text = element_blank()
  )
```

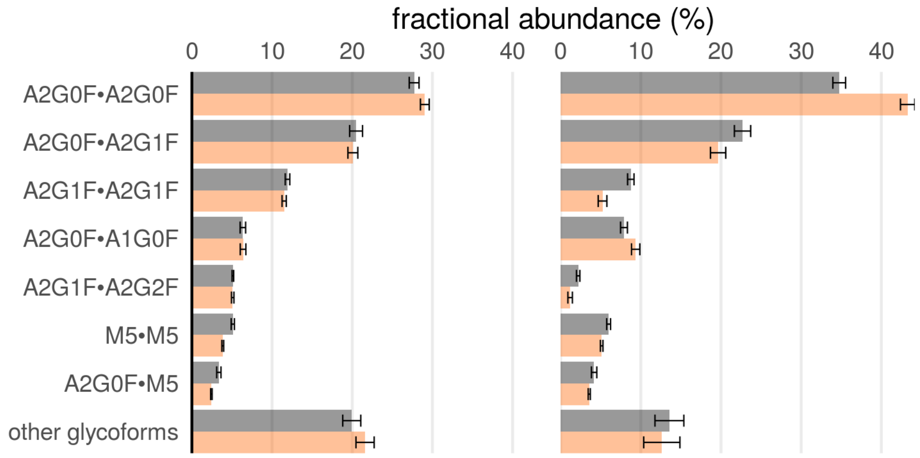


```
ggsave_default("figure6b", width = 12, height = 6)
```

c) Mass spectra of PNGase F-digested protein

Load mass spectra of both batches of PNGase F-digested denosumab and calculate zero-charge masses for charge state 53+.

```
glycation_spectrum_old <-
  load_mass_spectrum("denosumab/old/mass_spectrum_glycation.csv", "old")

glycation_spectrum_new <-
  load_mass_spectrum("denosumab/new/mass_spectrum_glycation.csv", "new")

glycation_spectra <-
  bind_rows(glycation_spectrum_old, glycation_spectrum_new) %>%
  mutate(batch = as_factor(batch))

glycation_zero_charge_spectra <-
  glycation_spectra %>%
  deconvolute(53L, 144000, 146000)
```

Draw a line chart (abundance vs. m/z) including a secondary x-axis with the zero-charge mass corresponding to the m/z values.

```
ggplot(glycation_zero_charge_spectra, aes(mz, abundance)) +
  geom_area(
    aes(fill = batch),
    show.legend = FALSE
  ) +
  geom_line(
```

```
    aes(color = batch),
    show.legend = TRUE,
    size = .25
  ) +
  annotate(
    "text",
    x = mass_to_mz(144200, 53),
    y = 87.5,
    label = "53+",
    fontface = "bold"
  ) +
  scale_color_manual(values = c("black", "#ff6600")) +
  scale_fill_manual(values = c("gray95", NA)) +
  scale_x_continuous(
    name = "m/z",
    sec.axis = sec_axis(
      ~mz_to_mass(., 53) / 1000,
      name = "mass (kDa)",
      breaks = c(144, 145, 146)
    )
  ) +
  scale_y_continuous(
    name = "relative abundance (%)",
    breaks = c(0, 50, 100),
    limits = c(-1, 100),
    expand = c(0, 0)
  ) +
  theme_bw() +
  theme(
    axis.line.x.top = element_line(),
    axis.line.y.left = element_line(),
    legend.background = element_blank(),
    legend.position = c(.8, .5),
    panel.border = element_blank(),
    panel.grid = element_blank(),
    plot.margin = margin(1.5, .1, .1, .1, "mm"),
    strip.text = element_blank()
  )
```

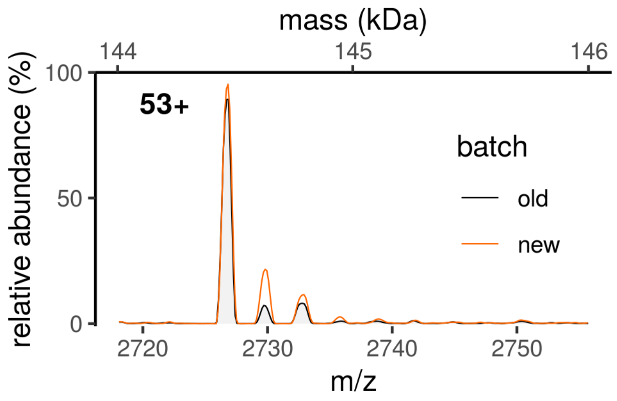


```r
ggsave_default("figure6c", width = 8, height = 5.4)
```

d) Abundance differences

The data frame containing CAFOG results for the denosumab batches currently has the following format:

composition	type	batch	abundance	error
0/0/0/0	observed	new	0.00	0.00
0/0/0/0	actual	new	0.00	0.00
0/0/0/0	observed	old	0.00	0.00
0/0/0/0	actual	old	0.00	0.00
0/10/4/0	observed	new	3.85	0.11
0/10/4/0	actual	new	5.12	0.16
0/10/4/0	observed	old	5.10	0.20
0/10/4/0	actual	old	5.99	0.24

Spread to a wide data frame.

```r
df_wide <-
  df_mab2 %>%
  select(-error) %>%
  pivot_wider(names_from = type, values_from = abundance) %>%
  unite(observed, actual, col = "observed_actual") %>%
  pivot_wider(names_from = batch, values_from = observed_actual) %>%
  separate(
    old,
    into = c("old_observed", "old_actual"),
    sep = "_",
    convert = TRUE
```

```
  ) %>%
  separate(
    new,
    into = c("new_observed", "new_actual"),
    sep = "_",
    convert = TRUE
  )
```

composition	old_observed	old_actual	new_observed	new_actual
2/6/8/0	27.72	34.74	29.05	43.27
2/7/8/0	20.48	22.69	20.08	19.64

Calculate observed/actual abundance differences between the old and new batch. Only include glycoforms with non-zero abundance.

```
df_diff <-
  df_wide %>%
  filter_if(is.numeric, any_vars(!near(., 0))) %>%
  mutate(
    observed = abs(old_observed - new_observed),
    actual = abs(old_actual - new_actual)
  ) %>%
  select(observed, actual, composition) %>%
  pivot_longer(
    c(observed, actual),
    names_to = "type",
    values_to = "difference"
  ) %>%
  mutate(type = as_factor(type))
```

The highest actual abundance differences between the old and new batch are:

```
df_diff %>%
  pivot_wider(names_from = type, values_from = difference) %>%
  top_n(5, actual) %>%
  arrange(desc(actual)) %>%
  mutate(composition = recode(composition, !!!glycoform_names)) %>%
  knitr::kable(digits = 1)
```

composition	observed	actual
A2G0F • A2G0F	1.3	8.5
A2G1F • A2G1F	0.4	3.5
A2G0F • A2G1F	0.4	3.0
A1G0F • A2G1F	0.0	1.5
A2G0F • A1G0F	0.0	1.5

Draw a scatter plot (abundance differences in percentage points vs. observed/actual) and connect corresponding points by lines.

```
ggplot(df_diff, aes(type, difference)) +
  geom_line(
    aes(group = composition),
    alpha = .1,
    size = .4,
    position = position_dodge(0.2)
  ) +
  geom_point(
    aes(color = type, group = composition),
    alpha = .3,
    position = position_dodge(0.2),
    show.legend = FALSE
  ) +
  scale_y_continuous(
    name = "abundance difference\nbetween batches (pp)",
    breaks = c(0, 5, 10),
    minor_breaks = NULL
  ) +
  xlab("") +
  scale_color_manual(values = RdBu) +
  coord_cartesian(ylim = c(0, 10.5)) +
  theme_bw() +
  theme(
    axis.ticks = element_blank(),
    panel.border = element_blank(),
    panel.grid.major.x = element_blank(),
    plot.margin = margin(.1, .1, .1, .1, "mm")
  )
```

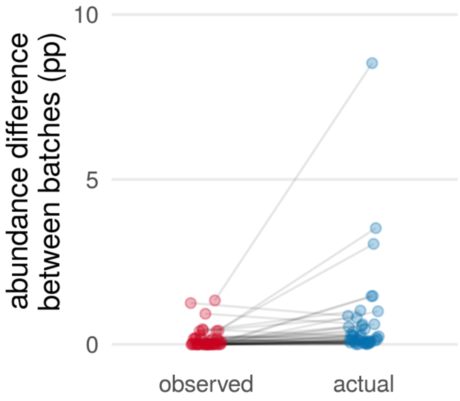


```
ggsave_default("figure6d", width = 6, height = 6)
```

e) Comparison to abundances simulated from released *N*-glycan data

Load glycan abundances as determined by released *N*-glycan analysis. (Unlike above, monosaccharide compositions are given as Hex/HexNAc/Fuc/Neu5Ac below.)

```
released_glycans <- read_csv("denosumab/released_glycans.csv")
```

composition	old	new
3/2/0/0	0.0	0.4
3/3/0/0	1.3	0.9
3/3/1/0	1.7	2.7
3/4/0/0	3.3	4.6
3/4/1/0	57.7	61.9
4/3/0/0	0.0	0.3

Simulate glycoform abundances assuming random pairing of glycans:

(a) Calculate the Cartesian square of the set comprising all detected released glycans.
(b) Multiply the abundances associated with the glycans in each 2-tuple.
(c) Aggregate these products for glycoforms with equal monosaccharide composition.
(d) Normalize the total simulated abundance to a value of 100.

```
simulated_abundances <-
  released_glycans %>%
  pull(composition) %>%
  crossing(x = ., y = .) %>%  # (a)
```

```r
  left_join(released_glycans, by = c(x = "composition")) %>%
  left_join(released_glycans, by = c(y = "composition")) %>%
  separate(
    x,
    into = c("Hex_x", "HexNAc_x", "Fuc_x", "Neu5Ac_x"),
    sep = "/",
    convert = TRUE
  ) %>%
  separate(
    y,
    into = c("Hex_y", "HexNAc_y", "Fuc_y", "Neu5Ac_y"),
    sep = "/",
    convert = TRUE
  ) %>%
  transmute(
    Hex = Hex_x + Hex_y,
    HexNAc = HexNAc_x + HexNAc_y,
    Fuc = Fuc_x + Fuc_y,
    Neu5Ac = Neu5Ac_x + Neu5Ac_y,
    old = old.x * old.y,  # (b)
    new = new.x * new.y
  ) %>%
  group_by(Hex, HexNAc, Fuc, Neu5Ac) %>%
  summarise_all(sum) %>%  # (c)
  ungroup() %>%
  mutate_if(is.double, ~ . / sum(.) * 100)  # (d)
```

Load CAFOG results for the two denosumab batches and calculate absolute deviations of observed/actual glycoform abundances from simulated abundances.

```r
batch <- c("old", "new")

deviations <-
  batch %>%
  map(~read_csv(str_glue("denosumab/{.}/results.csv"))) %>%
  set_names(batch) %>%
  bind_rows(.id = "batch") %>%
  select(batch, Hex, HexNAc, Fuc, Neu5Ac, abundance, corr_abundance) %>%
  mutate_all(~replace(., is.na(.), 0)) %>%
  left_join(
    simulated_abundances,
    by = c("Hex", "HexNAc", "Fuc", "Neu5Ac")
  ) %>%
```

```
  mutate(
    sim_abundance = case_when(
      batch == "old" ~ old,
      TRUE           ~ new
    )
  ) %>%
  select(-old, -new) %>%
  replace_na(list(sim_abundance = 0)) %>%
  filter(
    !near(abundance, 0) |
    !near(corr_abundance, 0) |
    !near(sim_abundance, 0)
  ) %>%
  unite(Hex:Neu5Ac, col = "composition", sep = "/") %>%
  mutate(
    observed = abs(sim_abundance - abundance),
    actual = abs(sim_abundance - corr_abundance)
  )
```

For each batch, the figure should highlight the five glycoforms for which CAFOG correction leads to the largest decrease in their absolute deviation from simulated abundance (`deviations_top5`). The largest decrease should be indicated separately (`deviations_max`).

```
tidy_deviations <- function(df) {
  df %>%
    pivot_longer(
      c(observed, actual),
      names_to = "delta",
      values_to = "value"
    ) %>%
    mutate(
      weight = case_when(
        delta == "observed" ~ abundance,
        TRUE                ~ corr_abundance
      )
    ) %>%
    select(-abundance:-sim_abundance)
}

deviations_top5 <-
  deviations %>%
  group_by(batch) %>%
  top_n(5, observed - actual) %>%
```

```r
  tidy_deviations()

deviations_max <-
  deviations %>%
  group_by(batch) %>%
  top_n(1, observed - actual) %>%
  tidy_deviations()
```

In addition, the background of the figure will show all data on changes in absolute deviation.

```r
deviations_all <-
  deviations %>%
  tidy_deviations()
```

Draw a scatter plot (absolute deviation from simulated abundance in percentage points vs. observed/actual) and connect corresponding points by lines. Highlight data as explained above and facet by batch. Point areas are proportional to the relative abundance of the respective glycoform.

```r
ggplot(deviations_all,
       aes(factor(delta) %>% fct_rev(), value, group = composition)) +
  geom_line(aes(color = batch), size = .4) +
  geom_point(
    aes(color = batch, size = weight),
    shape = 16
  ) +
  geom_line(data = deviations_top5) +
  geom_line(data = deviations_max, size = 1) +
  geom_point(
    data = deviations_top5,
    aes(color = delta, size = weight),
    shape = 16
  ) +
  scale_y_continuous(
    name = "absolute deviation from\nsimulated abundance (pp)",
    breaks = c(0, 5, 10),
    minor_breaks = NULL
  ) +
  scale_x_discrete(
    name = "",
    expand = expansion(add = .2),
    labels = c("obs.", "actual")
```

```
  ) +
  scale_color_manual(
    values = c(
      "#0571b0",                # actual
      alpha("#dbc9bd", 0.5),    # new
      "#ca0020",                # observed
      alpha("gray80", 0.5)      # old
    ),
    guide = FALSE
  ) +
  scale_size_area(guide = FALSE) +
  coord_cartesian(ylim = c(0, 10.5)) +
  facet_wrap(vars(factor(batch) %>% fct_rev())) +
  theme_bw() +
  theme(
    axis.ticks = element_blank(),
    panel.border = element_blank(),
    panel.grid.major.x = element_blank(),
    plot.margin = margin(.1, .1, .1, .1, "mm"),
    strip.background = element_blank(),
    strip.text = element_blank()
  )
```

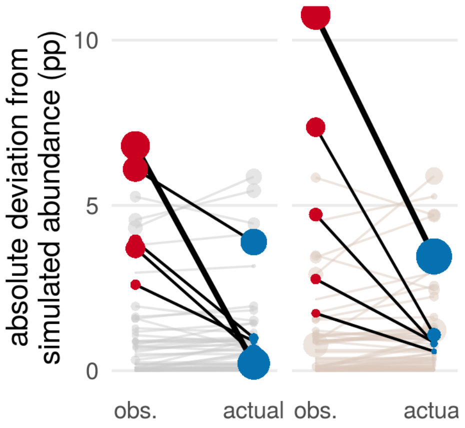


```
ggsave_default("figure6e", width = 6, height = 6)
```

The five glycoforms with the largest decrease in absolute deviation are:

batch	glycoform	observed absolute deviation
old	A2G0F • A2G0F	6.81
old	A2G0F • A2G1F	6.10
old	A2G1F • A2G2F	3.91
old	A2G1F • A2G1F	3.70
old	A2G2F • A2G2F	2.60
new	A2G0F • A2G0F	10.76
new	A2G1F • A2G1F	7.37
new	A2G1F • A2G2F	4.72
new	A2G2F • A2G2F	2.77
new	A2G2F • A2G2F+1Hex	1.74

The final figure will include root-mean-square deviations for all and the top 5 deviations.

```
bind_rows(
    all = deviations,
    top5 =
      deviations %>%
      group_by(batch) %>%
      top_n(5, observed - actual),
    .id = "selection"
) %>%
  group_by(selection, batch) %>%
  summarise(
    observed = sqrt(sum(observed^2) / n()),
    actual = sqrt(sum(actual^2) / n())
  ) %>%
  arrange(selection, desc(batch)) %>%
  knitr::kable(digits = 2)
```

selection	batch	observed	actual
all	old	2.07	1.69
all	new	2.26	1.70
top5	old	4.88	1.86
top5	new	6.37	1.72

Session info

This section contains detailed information about the platform and packages that have been used for compiling the analyses.

```
devtools::session_info()
#> - Session info ---------------------------------------------------------------
#>  setting  value
#>  version  R version 3.6.3 (2020-02-29)
#>  os       Linux Mint 18.1
#>  system   x86_64, linux-gnu
#>  ui       X11
#>  language (EN)
#>  collate  en_US.UTF-8
#>  ctype    en_US.UTF-8
#>  tz       Europe/Vienna
#>  date     2020-05-19
#>
#> - Packages -------------------------------------------------------------------
#>  package     * version date       lib source
#>  assertthat    0.2.1   2019-03-21 [1] CRAN (R 3.6.0)
#>  backports     1.1.5   2019-10-02 [1] CRAN (R 3.6.1)
#>  broom       * 0.5.5   2020-02-29 [1] CRAN (R 3.6.2)
#>  callr         3.4.3   2020-03-28 [1] CRAN (R 3.6.2)
#>  cellranger    1.1.0   2016-07-27 [1] CRAN (R 3.6.0)
#>  cli           2.0.2   2020-02-28 [1] CRAN (R 3.6.2)
#>  codetools     0.2-16  2018-12-24 [4] CRAN (R 3.5.2)
#>  colorspace    1.4-1   2019-03-18 [1] CRAN (R 3.6.0)
#>  crayon        1.3.4   2017-09-16 [1] CRAN (R 3.6.0)
#>  DBI           1.1.0   2019-12-15 [1] CRAN (R 3.6.1)
#>  dbplyr        1.4.2   2019-06-17 [1] CRAN (R 3.6.0)
#>  desc          1.2.0   2018-05-01 [1] CRAN (R 3.6.0)
#>  devtools      2.2.2   2020-02-17 [1] CRAN (R 3.6.2)
#>  digest        0.6.25  2020-02-23 [1] CRAN (R 3.6.2)
#>  dplyr       * 0.8.5   2020-03-07 [1] CRAN (R 3.6.2)
#>  ellipsis      0.3.0   2019-09-20 [1] CRAN (R 3.6.1)
#>  evaluate      0.14    2019-05-28 [1] CRAN (R 3.6.0)
#>  fansi         0.4.1   2020-01-08 [1] CRAN (R 3.6.2)
#>  farver        2.0.3   2020-01-16 [1] CRAN (R 3.6.1)
#>  forcats     * 0.5.0   2020-03-01 [1] CRAN (R 3.6.2)
#>  fs          * 1.3.2   2020-03-05 [1] CRAN (R 3.6.2)
#>  gdtools     * 0.2.1   2019-10-14 [1] CRAN (R 3.6.1)
#>  generics      0.0.2   2018-11-29 [1] CRAN (R 3.6.0)
#>  ggplot2     * 3.3.0   2020-03-05 [1] CRAN (R 3.6.2)
```

```
#>  glue          1.3.2   2020-03-12 [1] CRAN (R 3.6.2)
#>  gtable        0.3.0   2019-03-25 [1] CRAN (R 3.6.0)
#>  haven         2.2.0   2019-11-08 [1] CRAN (R 3.6.1)
#>  highr         0.8     2019-03-20 [1] CRAN (R 3.6.0)
#>  hms           0.5.3   2020-01-08 [1] CRAN (R 3.6.1)
#>  htmltools     0.4.0   2019-10-04 [1] CRAN (R 3.6.1)
#>  httr          1.4.1   2019-08-05 [1] CRAN (R 3.6.0)
#>  jsonlite      1.6.1   2020-02-02 [1] CRAN (R 3.6.1)
#>  knitr         1.28    2020-02-06 [1] CRAN (R 3.6.1)
#>  labeling      0.3     2014-08-23 [1] CRAN (R 3.6.0)
#>  lattice       0.20-41 2020-04-02 [4] CRAN (R 3.6.3)
#>  lifecycle     0.2.0   2020-03-06 [1] CRAN (R 3.6.2)
#>  lubridate     1.7.4   2018-04-11 [1] CRAN (R 3.6.0)
#>  magrittr      1.5     2014-11-22 [1] CRAN (R 3.6.0)
#>  memoise       1.1.0   2017-04-21 [1] CRAN (R 3.6.0)
#>  modelr        0.1.6   2020-02-22 [1] CRAN (R 3.6.2)
#>  munsell       0.5.0   2018-06-12 [1] CRAN (R 3.6.0)
#>  nlme          3.1-147 2020-04-13 [4] CRAN (R 3.6.3)
#>  pillar        1.4.3   2019-12-20 [1] CRAN (R 3.6.1)
#>  pkgbuild      1.0.6   2019-10-09 [1] CRAN (R 3.6.1)
#>  pkgconfig     2.0.3   2019-09-22 [1] CRAN (R 3.6.1)
#>  pkgload       1.0.2   2018-10-29 [1] CRAN (R 3.6.0)
#>  prettyunits   1.1.1   2020-01-24 [1] CRAN (R 3.6.1)
#>  processx      3.4.2   2020-02-09 [1] CRAN (R 3.6.1)
#>  ps            1.3.2   2020-02-13 [1] CRAN (R 3.6.2)
#>  purrr       * 0.3.3   2019-10-18 [1] CRAN (R 3.6.1)
#>  R6            2.4.1   2019-11-12 [1] CRAN (R 3.6.1)
#>  Rcpp          1.0.4   2020-03-17 [1] CRAN (R 3.6.2)
#>  readr       * 1.3.1   2018-12-21 [1] CRAN (R 3.6.0)
#>  readxl        1.3.1   2019-03-13 [1] CRAN (R 3.6.0)
#>  remotes       2.1.1   2020-02-15 [1] CRAN (R 3.6.2)
#>  reprex        0.3.0   2019-05-16 [1] CRAN (R 3.6.0)
#>  rlang         0.4.5   2020-03-01 [1] CRAN (R 3.6.2)
#>  rmarkdown     2.1     2020-01-20 [1] CRAN (R 3.6.1)
#>  rprojroot     1.3-2   2018-01-03 [1] CRAN (R 3.6.0)
#>  rstudioapi    0.11    2020-02-07 [1] CRAN (R 3.6.1)
#>  rvest         0.3.5   2019-11-08 [1] CRAN (R 3.6.1)
#>  scales        1.1.0   2019-11-18 [1] CRAN (R 3.6.1)
#>  sessioninfo   1.1.1   2018-11-05 [1] CRAN (R 3.6.0)
#>  stringi       1.4.6   2020-02-17 [1] CRAN (R 3.6.2)
#>  stringr     * 1.4.0   2019-02-10 [1] CRAN (R 3.6.0)
#>  svglite       1.2.3   2020-02-07 [1] CRAN (R 3.6.1)
#>  systemfonts   0.1.1   2019-07-01 [1] CRAN (R 3.6.1)
```

```
#>  testthat      2.3.2   2020-03-02 [1] CRAN (R 3.6.2)
#>  tibble      * 3.0.0   2020-03-30 [1] CRAN (R 3.6.2)
#>  tidyr       * 1.0.2   2020-01-24 [1] CRAN (R 3.6.1)
#>  tidyselect    1.0.0   2020-01-27 [1] CRAN (R 3.6.1)
#>  tidyverse   * 1.3.0   2019-11-21 [1] CRAN (R 3.6.1)
#>  usethis       1.5.1   2019-07-04 [1] CRAN (R 3.6.0)
#>  vctrs         0.2.4   2020-03-10 [1] CRAN (R 3.6.2)
#>  viridisLite   0.3.0   2018-02-01 [1] CRAN (R 3.6.0)
#>  withr         2.1.2   2018-03-15 [1] CRAN (R 3.6.0)
#>  xfun          0.12    2020-01-13 [1] CRAN (R 3.6.1)
#>  xml2          1.2.5   2020-03-11 [1] CRAN (R 3.6.2)
#>  yaml          2.2.1   2020-02-01 [1] CRAN (R 3.6.1)
#>
#> [1] /home/wolfgang/Programme/R/3.6
#> [2] /usr/local/lib/R/site-library
#> [3] /usr/lib/R/site-library
#> [4] /usr/lib/R/library
```

References

[1] S. van der Walt, S. C. Colbert, G. Varoquaux, *Comput. Sci. Eng.* **2011**, *13*, 22–30.

[2] W. McKinney, in *Proceedings of the 9th Python in Science Conference* (Eds.: S. van der Walt, J. Millman), **2010**, pp. 51–56.

[3] A. A. Hagberg, D. A. Schult, P. J. Swart, in *Proceedings of the 7th Python in Science Conference* (Eds.: G. Varoquaux, T. Vaught, J. Millman), **2008**, pp. 11–15.

[4] C. Regl, T. Wohlschlager, W. Esser-Skala, I. Wagner, M. Samonig, J. Holzmann, C. G. Huber, *mAbs* **2019**, *11*, 569–582.

[5] N. Hulstaert, J. Shofstahl, T. Sachsenberg, M. Walzer, H. Barsnes, L. Martens, Y. Perez-Riverol, *J. Proteome Res.* **2020**, *19*, 537–542.

[6] Y. Perez-Riverol, A. Csordas, J. Bai, M. Bernal-Llinares, S. Hewapathirana, D. J. Kundu, A. Inuganti, J. Griss, G. Mayer, M. Eisenacher, et al., *Nucleic Acids Res.* **2018**, *47*, D442–D450.

[7] S. Neelamegham, K. Aoki-Kinoshita, E. Bolton, M. Frank, F. Lisacek, T. Lütteke, N. O'Boyle, N. H. Packer, P. Stanley, P. Toukach, et al., *Glycobiology* **2019**, *29*, 620–624.

[8] Z. Zhang, *Anal. Chem.* **2009**, *81*, 8354–8364.

[9] Y. Shafranovich, *Common Format and Mime Type for Comma-Separated Values (Csv) Files*, RFC Editor; Internet Requests For Comments; RFC Editor, **2005**.

[10] Y. Xie, J. Allaire, G. Grolemund, *R Markdown: The Definitive Guide*, Chapman; Hall/CRC, Boca Raton, Florida, **2018**.

[11] Y. Xie, *Dynamic Documents with R and Knitr*, Chapman; Hall/CRC, Boca Raton, Florida, **2015**.

[12] R Core Team, *R: A Language and Environment for Statistical Computing*, R Foundation For Statistical Computing, Vienna, Austria, **2018**.

[13] H. Wickham, M. Averick, J. Bryan, W. Chang, L. McGowan, R. François, G. Grolemund, A. Hayes, L. Henry, J. Hester, et al., *J. Open Source Softw.* **2019**, *4*, 1686.

[14] M. A. Harrower, C. A. Brewer, *Cartogr. J.* **2003**, *40*, 27–37.

[15] T. Coplen, J. Böhlke, P. D. Bievre, T. Ding, N. Holden, J. Hopple, H. Krouse, A. Lamberty, H. Peiser, K. Revesz, et al., *Pure Appl. Chem.* **2002**, *74*, 1987–2017.
